# Short-term microbial effects of a large-scale mine-tailing storage facility collapse on the local natural environment

**DOI:** 10.1371/journal.pone.0196032

**Published:** 2018-04-25

**Authors:** Heath W. Garris, Susan A. Baldwin, Jon Taylor, David B. Gurr, Daniel R. Denesiuk, Jonathan D. Van Hamme, Lauchlan H. Fraser

**Affiliations:** 1 Departments of Natural Resource Sciences & Biological Sciences, Thompson Rivers University, Kamloops, British Columbia, Canada; 2 Department of Chemical and Biological Engineering, University of British Columbia, Vancouver, British Columbia, Canada; Universita degli Studi di Milano-Bicocca, ITALY

## Abstract

We investigated the impacts of the Mount Polley tailings impoundment failure on chemical, physical, and microbial properties of substrates within the affected watershed, comprised of 70 hectares of riparian wetlands and 40 km of stream and lake shore. We established a biomonitoring network in October of 2014, two months following the disturbance, and evaluated riparian and wetland substrates for microbial community composition and function via 16S and full metagenome sequencing. A total of 234 samples were collected from substrates at 3 depths and 1,650,752 sequences were recorded in a geodatabase framework. These data revealed a wealth of information regarding watershed-scale distribution of microbial community members, as well as community composition, structure, and response to disturbance. Substrates associated with the impact zone were distinct chemically as indicated by elevated pH, nitrate, and sulphate. The microbial community exhibited elevated metabolic capacity for selenate and sulfate reduction and an abundance of chemolithoautotrophs in the *Thiobacillus thiophilus/T*. *denitrificans/T*. *thioparus* clade that may contribute to nitrate attenuation within the affected watershed. The most impacted area (a 6 km stream connecting two lakes) exhibited 30% lower microbial diversity relative to the remaining sites. The tailings impoundment failure at Mount Polley Mine has provided a unique opportunity to evaluate functional and compositional diversity soon after a major catastrophic disturbance to assess metabolic potential for ecosystem recovery.

## Introduction

Mining produces large quantities of waste material annually, and containment failures present challenges to water quality and wildlife. Contemporary mining involves extensive restructuring of landscapes where vegetation and naturally-accreted soils are removed, and contiguous bedrock is exposed and partially converted to coarse and fine wastes. Depending on the parent material composition, these wastes are prone to accelerated chemical weathering, constituting a fundamentally altered biogeochemical setting by comparison with the pre-existing ecosystem [1,2]. Fine wastes (often termed ‘tailings’) typically present the greatest challenges for containment as they have the greatest potential for leaching (due to high surface area for chemical weathering) and mobility as dust or in fluid suspensions. For this reason, current best practices of mining operations worldwide manage tailings to be physically and metabolically isolated from the surrounding watershed through containment in waste impoundments, such as tailings storage facilities [3]. Over the duration of a mine’s active lifetime, the quantity of mine wastes accumulated on site can become immense. For example, before the impoundment failure at the Mount Polley Mine (“the Mine”), British Columbia, Canada, in 2014, the Mine’s tailings storage facility had the capacity to contain 74 million cubic meters of hydrated tailings within an impoundment occupying 2 km^2^ [4].

Despite best practices for construction, inspection and maintenance of tailing storage facilities, containment failures have occurred [5]. Under these circumstances, uncontrolled deposition into previously protected surroundings disturbs physical and chemical properties, and their associated above- and below-ground ecosystems within the surrounding landscape. While surface erosion and deposition are clearly observed, sub-surface disruptions are more challenging to observe and/or quantify. Sudden major disturbances in the physical, chemical and biological structures of natural soils and sediments are likely to impact essential ecosystem functions such as nutrient transport [6], weathering and erosion, chemical detoxification, water quality preservation [7], support of primary productivity [8] and resilience to perturbations [9]. These processes will determine the potential for recovery in impacted areas. It follows that assessment of the affected soils and sediments soon after the disturbance is required to evaluate the degree to which these areas resisted the impact and in what ways their ecosystem functions may have been altered.

Due to their topographic position within landscapes, wetlands and riparian areas are often the first recipients of mined substrates and chemicals when containment failures occur. Owing to their relatively high biological productivity and accumulation of soil organic matter, these systems are noted for their capacity to resist toxic inputs and have even been used for passive remediation of mine effluents [10,11]. Mine tailings consist of minerals that might become sources of metal and metalloid contaminants depending on the mineralogy of the ore body, mineral processing procedures and environmental conditions inside the tailings. Sequestration and detoxification of metals and metalloids is facilitated through metabolic processes carried out by specific types of microbes that inhabit niches found in organic-rich soils and sediments. Many mine remediation approaches capitalize on these natural metal sequestration processes by engendering environmental conditions conducive to growth of beneficial microbes, such as sulphate-reducing bacteria, Rhizobia and many other microbes with the capacity for metal immobilization [12–16].

During a catastrophic disturbance, such as a mine tailings impoundment failure, the aforementioned ecosystem functions of soil and sediment microbiomes are challenged. Few studies have evaluated the role soil microbial community composition and alpha/beta diversity play in resilience to disturbance [17–20]. Microbial communities may respond to sudden disturbances by exhibiting resilience enabling rapid recovery back to pre-existing ecosystem functions [21]. They may persist in a perpetually suppressed state, or exhibit shifts to alternate, less desirable states that prohibit restoration or reduce the beneficial relationships between microbial diversity, soil and plant quality, and ecosystem sustainability [22]. It is also possible that microbial communities may shift in response to sudden environmental changes towards establishment of alternate structural and functional states that promote restoration, to albeit alternate but stable ecosystems [23]. Deposition of tailings might result in chronic disturbance to soil and sediment microbiomes, which is thought to lead to reduced ecosystem resiliency should substrates be subjected to larger stressors in the future [24].

For this study, we addressed disturbance of the structure and function of riparian soil and sediment microbial communities in the path of dispersed tailings and in non-impacted sites two months following the failure of a tailings storage facility at Mount Polley Mine, (Likely, BC Canada). The impoundment failure produced a watershed-scale disturbance gradient, where soils were eroded to bedrock and replaced with tailings (characteristic of Hazeltine Creek), while others exhibited varying degrees of mixing and burial (characteristic of Polley Lake). As a result, Mount Polley is unique in the timeliness of baseline data collected following the disturbance, but shares the disadvantage of many major disturbance studies in the lack of data quantifying heterogeneity in system parameters before the disturbance occurred.

The Mount Polley Mine tailings storage facility experienced a failure of foundation material and subsequent breach at approximately 2:00 am, Monday, August 4, 2014 in central British Columbia, Canada [25]. Foundation failures represent ~6% of the 147 tailings impoundment failures reported globally [26]. Over the following 2–3 days approximately 10.6 million m^3^ of mine-influenced, untreated water and sediment escaped into the natural watershed [27], dispersing over approximately 25 hectares of lake-associated wetland and 45 hectares of riparian area, and depositing together with scoured natural material into Polley Lake and Quesnel Lake with the risk of further transportation downstream into the Quesnel River [4]. Impacts of this disturbance on soil and sediment microbial populations of the affected riparian area and lake-associated wetlands were determined through a metagenomic survey along the gradient of tailings deposition away from the impoundment failure origin. These metagenomic records were combined with physical and chemical characteristics of the collected substrates and analyzed within a geodatabase framework to assess microbial community connections to environmental variables including soil, nutrient, and water status, and passive uptake of contaminants.

## Materials & methods

### Sample site locations

**Fig 1:**
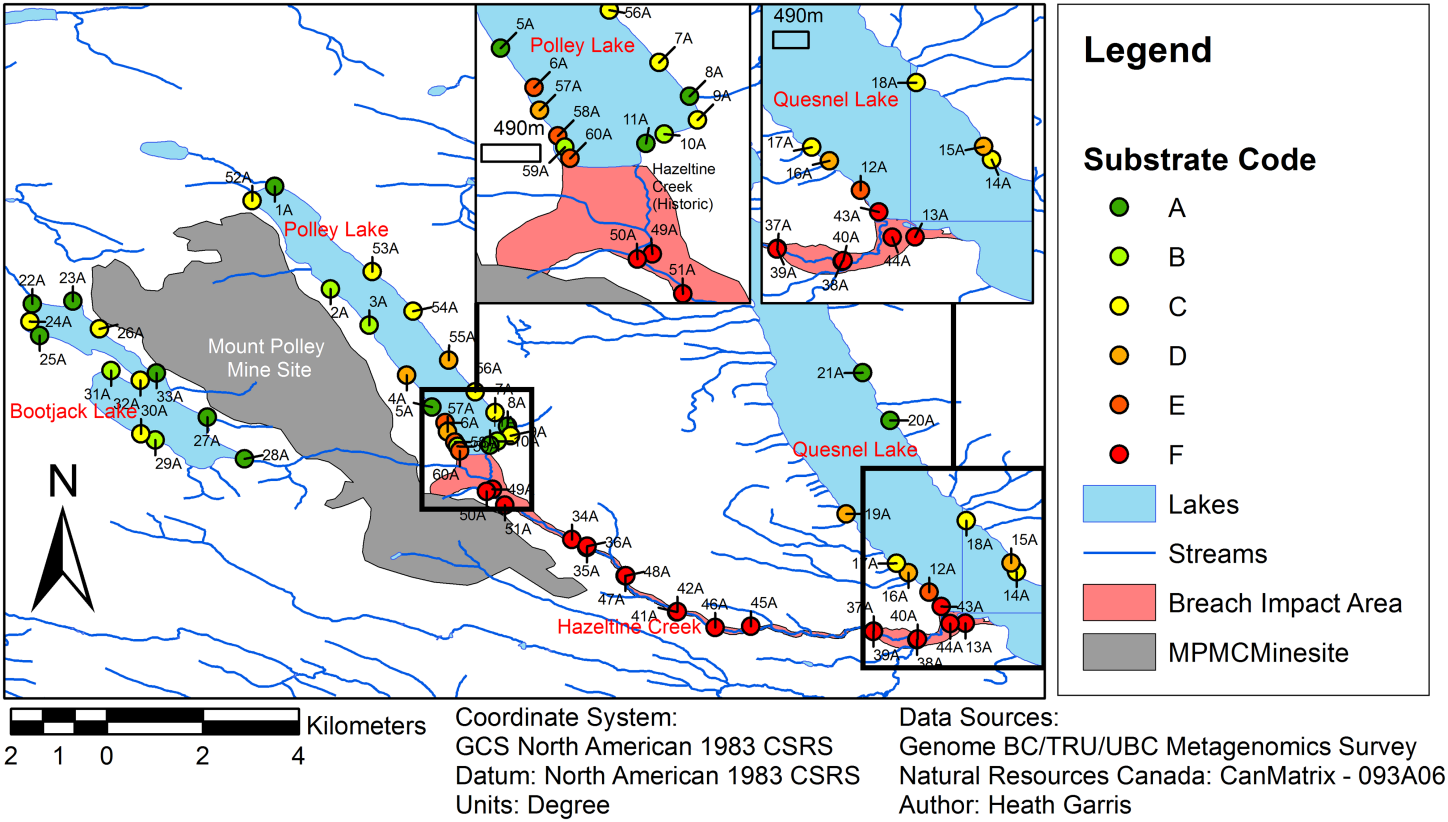
Biomonitoring network sample sites. Sites are located within the affected watershed, in hydric soils associated with existing water bodies. With the exception of Bootjack Lake (reference area) the selected sites reflect a continuum of physical and chemical impacts from the event. Substrate Code colours reflect designations detailed in Fig 2.

Field sampling was conducted with permission from Mount Polley Mine Corporation. Suitable locations for sample collection were determined by performing a survey of the affected watershed via high resolution aerial photography and Landsat Thematic Mapper Imagery [28]. Sampling consisted of 60 established field sites distributed within the Hazeltine Creek floodplain (19), Polley Lake floodplain (20), Quesnel Lake Floodplain (9), and the Bootjack Lake Floodplain (12) (Fig 1).

**Fig 2:**
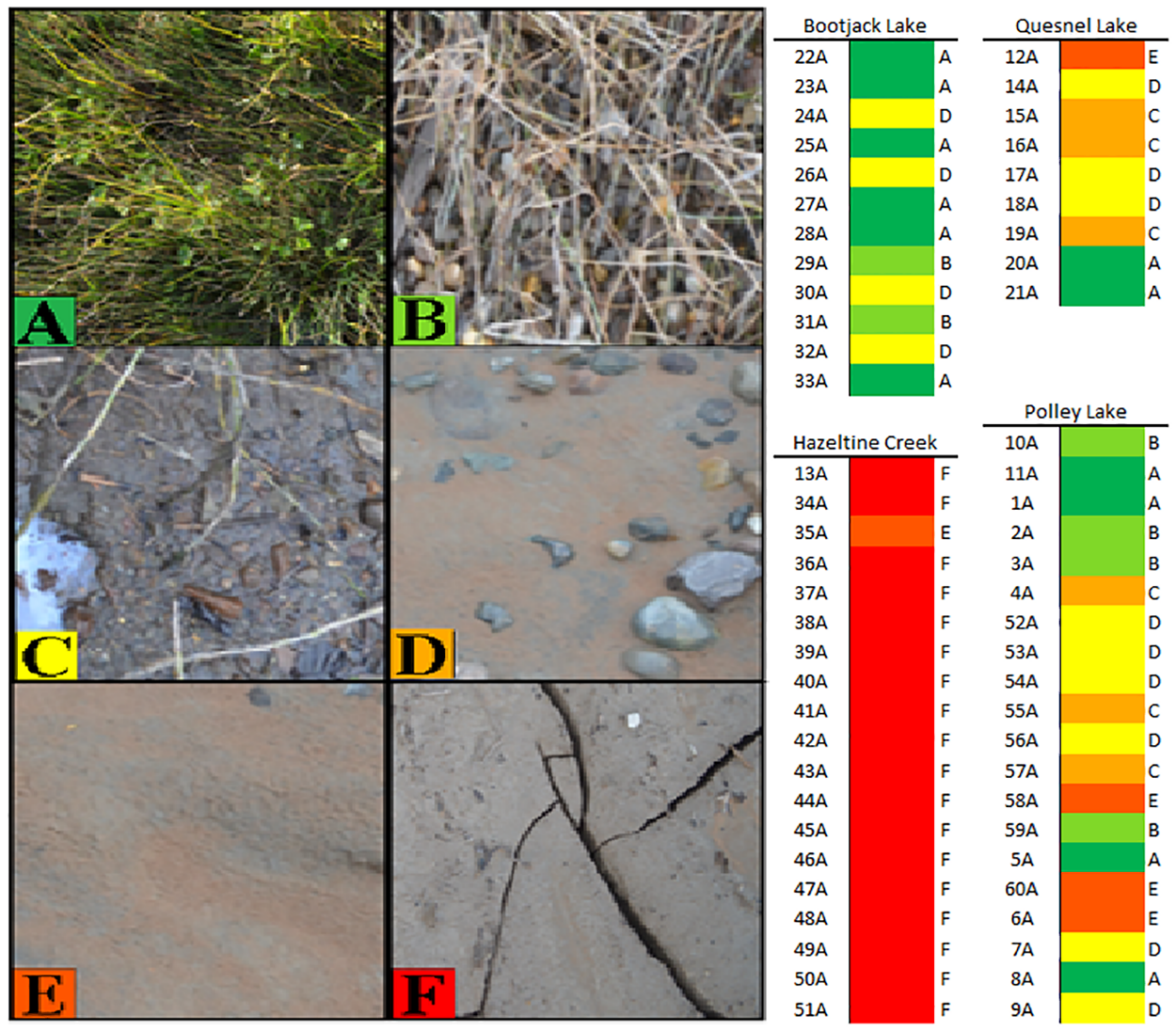
Samples were collected to represent the types of habitats affected by the spill including. (A) organic-rich soil supporting a diverse wetland community including living, senesced and decayed vegetation, (B) organic-dominated lakeshore lacking extensive living vegetation, (C) mineral lakeshore including exposed native bedrock, cobbles, clays or sands, (D) native substrates with evidence of tailings deposition (visible accretions of tailings sediments intermingled with native substrate or coating of plant stems/leaves with fine tailings), (E) burial of native substrate in coarse tailings sands, (F) replacement of native substrate with a mixture of fine tailings and sands. The table to the right presents mean surface material estimates (n = 5, 1 m^2^ quadrats) for sample sites with habitat classification (matching colors from figure to the left).

Shoreline ecosystems were targeted since primary macrophyte productivity is highest in these regions and consequently they are likely to harbour diverse below-ground microbial communities important for ecosystem function. For stream and lakeside sample sites, a base-point was established (marked via GPS (S1 Table), survey flagging, and a buried galvanized steel pin).

### Sample collection

**Fig 3:**
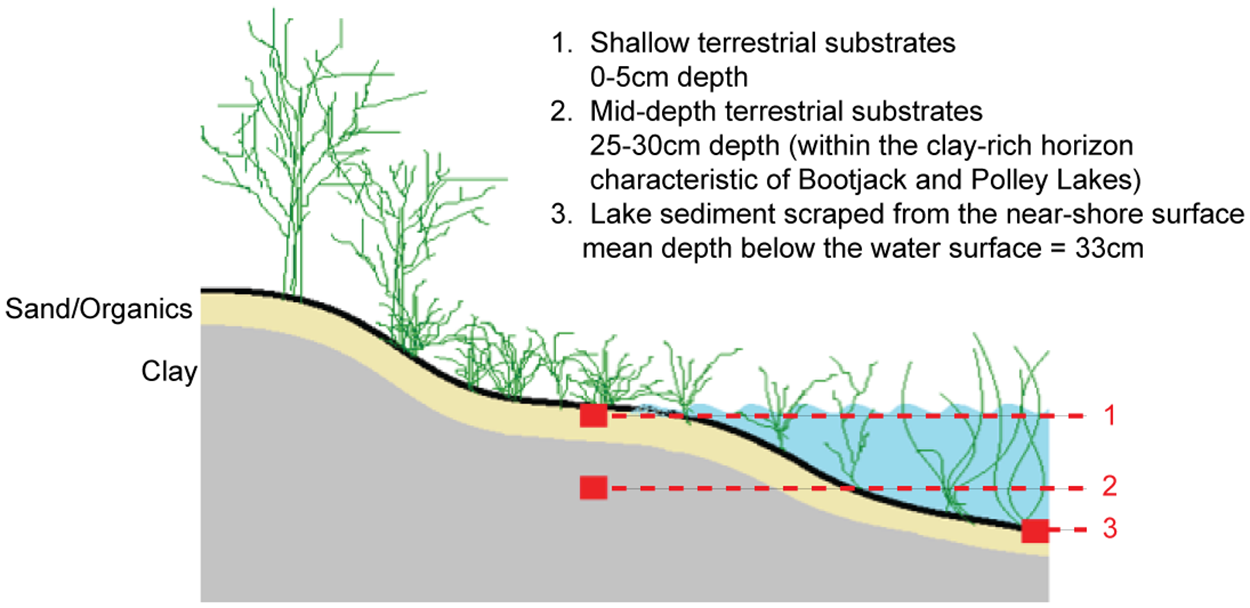
Cartoon cross-section of an idealized hydrosphere representing layers that were sampled.

Monitoring sites were sampled between October 7^th^ and 23^rd^, 2014. Two to three hundred grams of substrate were removed from 6 locations at each site and placed into sterile Whirl-Pak^®^ bags. Samples were collected from three distinct habitats (Fig 3).

**Fig 4:**
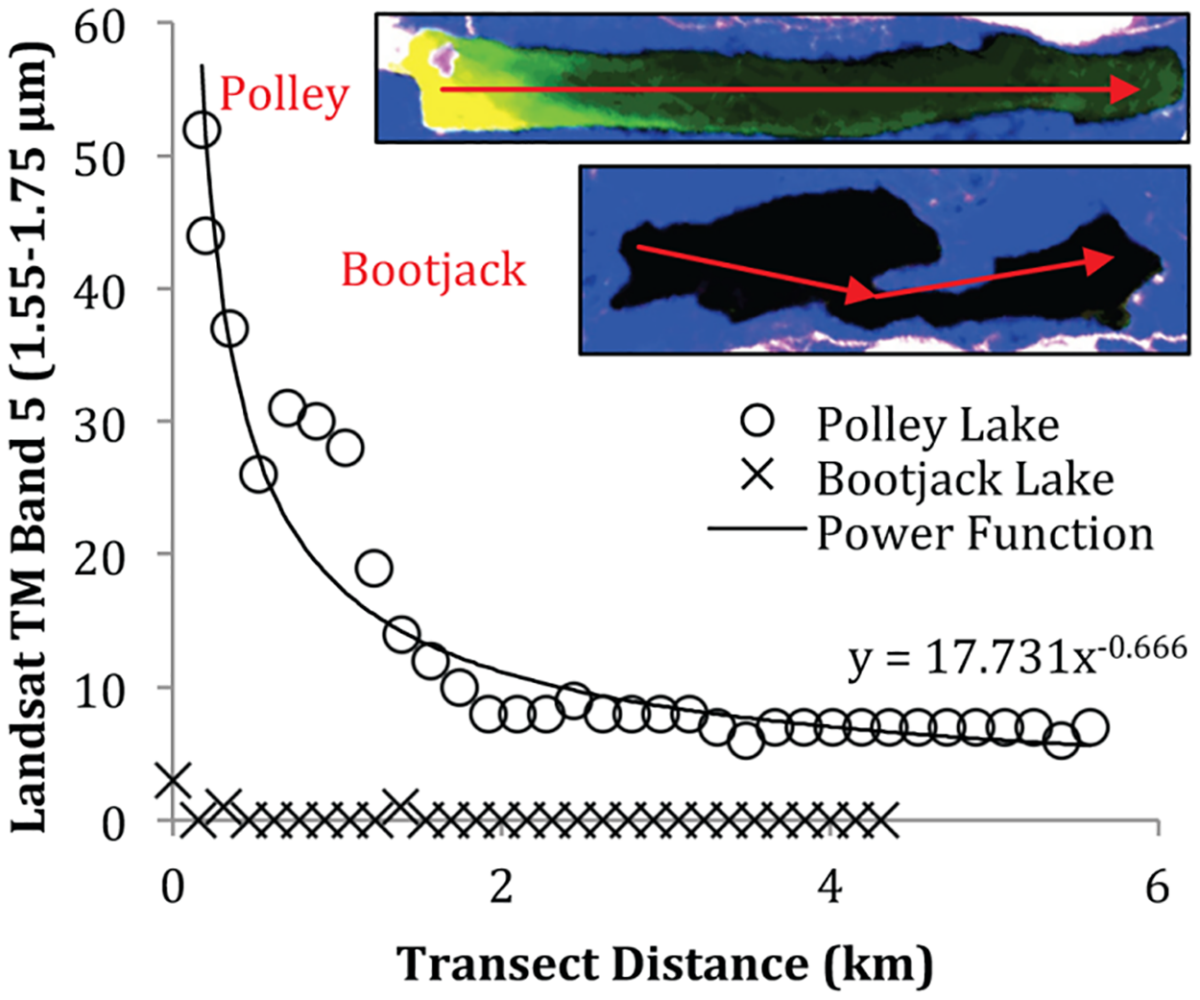
Comparison of short-wave infrared reflectance (represented in yellow-green) measured in Polley and Bootjack Lakes 48 hours post-dam failure visually (inset) and as a function of distance away from the impoundment failure.

The riparian and lake areas affected by the tailings impoundment failure included Hazeltine Creek, Polley Lake and Quesnel Lake (Fig 1). The extent and amount of tailings deposited terrestrially and into the lakes have been reported in detail in the Post Event Environmental Impact Assessment Report published after much survey work was conducted in the months following the event [4]. Less than 48 hours after the failure, short-wave infrared reflectance from Bootjack and Polley Lakes was recorded by Landsat [28](USGS 2011) and used to detect solids suspended in Polley Lake (Fig 4).

In comparison to Bootjack Lake, which was not in the path of the deposited tailings since it is located west of the mine site, higher loadings of suspended solids were clearly visible in Polley Lake. Based on the intensity of the reflectance, the amount of sediment attenuation into Polley Lake was estimated to follow a logarithmic rate law with distance away from the southeast shore of Polley Lake where the deposited tailings entered the Lake (Fig 4). Spacing of sampling locations along the shores of Polley Lake was based on this relationship (Fig 1).

Two samples were taken from the surficial layer of terrestrial substrate (0–5 cm depth) 3 m apart 1 m parallel to the water’s edge. Two samples were collected from the mid-depth terrestrial layer (25–30 cm depth) at the same locations using a soil probe or Edelman auger. For Bootjack and Polley Lakes these latter samples constituted clay-rich horizons. Two additional samples were removed from the sub-aqueous sediment layer within the permanently submerged area of the littoral zone. Pore and surface water samples were collected in separate bags from the sediment layer. Samples for sulphate and sulfide analysis were preserved in 0.5% zinc acetate. Samples were frozen on dry ice immediately after sampling and then stored at -80°C until processing. Plot photos were taken for each of the sample locations using a Nikon D300 digital SLR and included a meter stick and 1m X 1m grid placed in view for reference. These photos were used to qualitatively describe surface mineral composition/grain-size and amount/composition of vegetation cover (Fig 2). Habitats varied in their estimated degree of disturbance from no visible impact characteristic of Bootjack Lake to erosion of all native substrates and replacement with material from the tailings impoundment (characteristic of Hazeltine Creek). Multiple quantitative approaches were applied to determine distinctions between major substrate types (including prevailing surficial grain sizes and organic content). In practice, freshly deposited tailings were easily distinguished from native substrates by their light color and mixture of reddish-grey sands and fine, grey sediments.

### Physical and chemical parameters

Water quality parameters, pH, temperature, conductivity, dissolved oxygen (DO), oxidation-reduction potential (ORP), and turbidity were recorded in the field using a YSI EXO2 Multiparameter Water Quality SONDE (YSI International) for surface waters (33_±3_cm depth draft), adjacent to the shoreline where terrestrial samples were collected and for porewater immediately following extraction from augured holes. Later, in the laboratory, pore and surface water samples were thawed and analyzed for sulphate (APHA [29] turbidimetric method 4500-SO_4_^2-^ E), sulfide (methylene blue method 4500-S^2-^ D), ammonium (phenate method 4500-NH_3_ F), phosphate (ascorbic acid method 4500-P E) and total nitrite/nitrate-N (cadmium reduction method 4500-NO_3_^-^ E followed by colorimetric method 4500-NO_2_^-^ B). Physical and chemical metadata are available through DRYAD (datadryad.org; doi:10.5061/dryad.d52df21).

### Microbial community quantification

#### DNA extraction

Just prior to DNA extraction, sample bags with solids samples were thawed and the contents homogenized manually. Three 250 mg subsamples from each bag were taken for genomic DNA (gDNA) extraction using the Mo Bio PowerSoil^®^ DNA Isolation kit (Mo Bio Laboratories Inc., Carlsbad CA, USA). The three gDNA extractions were combined into a single tube. The concentration was determined using a Qubit^®^ 3.0 high sensitivity fluorometric double stranded DNA assay (ThermoFisher Scientific, Waltham, MA, USA).

#### Amplicon PCR, library preparation, and sequencing

Small sub-unit ribosomal DNA (SSU rDNA) amplicons were prepared for sequencing using procedures in the Illumina “16S Metagenomic Sequencing Library Preparation Guide” [30] with the following modifications: PCR reactions used the SsoFast^**™**^ EvaGreen^®^ Supermix (Bio-Rad Laboratories (Canada) Ltd., Mississauga, ON, Canada), and primers for the V6-V8 region were used: 926f 5’ AAACTYAAAKGAATTGRCGG and 1392r 5’ ACGGGCGGTGTGTRC 3’. After recording the concentration in each indexed 16S rDNA amplicon pool, samples were diluted to obtain 4 nM DNA in 20 μL of DI H_2_O. Paired-end 300bp sequencing was performed on an Illumina MiSeq at the University of British Columbia Sequencing Centre in the Faculty of Pharmaceutical Sciences using the Illumina MS-102-3003-MiSeq Reagent Kit v3 (600 cycles) and PhiX Control Kit v3. DNA for metagenomic whole genome sequencing was purified from the high molecular weight band on 1% agarose gel using excision and the QIAquick Gel Extraction Kit (Qiagen) and the extracted genomic DNA was quantified using Qubit HS DNA assay (Thermo-Fisher Scientific). Approximately 1ng total DNA was used for library preparation. A DNA fragment library with an index at each end was generated for all samples using the Nextera XT DNA sample preparation kit (Illumina catalog no. GA09115). For quality control and size evaluation, the sequencing library was analyzed on a Bioanalyzer 2100 (Agilent Technologies). Paired-end sequencing was performed as for the amplicon sequencing. Raw sequence files from both amplicon and whole genome sequencing were deposited in the sequence read archive (SRA) on http://www.ncbi.nlm.nih.gov under project accession number PRJNA433688.

#### Bioinformatics

Sequence quality was inspected using FastQC version 0.10.1 [31]. Since the quality of the forward reads was far superior to that for the reverse reads, only the forward reads were used for further analysis. The raw forward reads were quality filtered using the usearch [32] function fastq_filter to truncate to the end sequences if the Phred quality score was less than 15, remove sequences less than 150 bases in length, and to discard reads with expected errors greater than 0.5 and those reads with ambiguous base calls. High quality sequences were clustered into 97% sequence similarity cut-off operational taxonomic units (OTUs) using the usearch function cluster_otus. Taxonomic assignment to representative sequences from each OTU was performed with MOTHUR version 1.36.1 [33] function classify.seqs using as the reference the SILVA SSU database version 123 [34].

Whole genome sequence reads were preprocessed to remove Illumina and Nextera adapters, low quality (Q < 25) and short (length < 100 bases) reads using Trim Galore 0.4.1 (http://www.bioinformatics.babraham.ac.uk/projects/trim_galore/). High quality reads were assembled with velvet and meta-velvet [35]. Open reading frames (ORFs) were predicted from the assembled contigs using Prodigal version 1.0.1, translated to amino acid sequences and annotated using custom databases for respiratory denitrification proteins (nitrate reductase (NarG, NapA), nitrite reductase to NO (NirK, NirS), nitrite reductase to ammonia (NrfH) and nitrous oxide reductase (NosZ)), selenate reductases (SerABC, SrdA and YgfK), dimethylsulfoxide (DMSO) reductases [36], dissimilatory sulfite reductase (DsrAB) and the protein database Uniref90. All amino acid sequences for the custom databases, excluding the DMSO reductases, were downloaded from the Uniprot repository (http://www.uniprot.org/, accessed 3 July 2017). Open reading frame prediction and annotation were carried out using the Metapathways version 2.5.1 pipeline [37]. Only hits with bitscores equal to or greater than 80 were taken into consideration. Consistency between functional annotation using the denitrification, selenate reductase and sulfate reductase databases and the Uniref90 database was confirmed. Since selenate reductases SerAB and SrdA from *Thauera selenatis* and *Bacillus selenatarsenatis*, respectively, belong to the DMSO reductase family of molybdopterin oxidoreductases, ORFs with hits to SerAB and SrdA amino acid sequences were also queried against a custom DMSO database [36] to confirm their annotation to selenate reductase with the highest bitscore.

Microbial population diversity (richness and evenness) in each sample was assessed in terms of taxonomic richness (# of detected OTUs) and Simpson’s Diversity (1-D). The OTU table was subsampled with MOTHUR function sub.sample to 3,000 reads per sample and split into three OTU tables for each layer or hydrozone: surficial (0–5 cm), deep (25–30) cm and the aquatic sediments.

Beta-diversity of samples was explored on non-metric multidimensional scaling (NMDS) ordination plots produced using Bray-Curtis dissimilarity with the vegan package of tools in R version 3.3.1. Statistical comparisons of microbial population compositions were carried out using UniFrac distances [38] with MOTHUR functions unifrac_weighted and unifrac_unweighted. Indicator species for each biome (Bootjack Lake, Polley Lake, Quesnel Lake and Hazelton Creek) were determined using the method in Dufrêne and Legendre [39] for each hydrozone layer.

## Results & discussion

### Site characteristics & chemistry

Sites were classified into different habitats according to the surficial substrate appearance (Fig 2). Habitats A to C exhibited an array of macrophytes, organic deposits and minerals and little to no evidence of the presence of tailings. Grey tailings and magnetite sands presented a distinct visual contrast to the darker native substrates, making them easy to distinguish on the ground. Tailings at Mount Polley are derived from an alkalic porphyry copper gold deposit where copper and copper sulphides are removed prior to storage in the tailings impoundment [40]. Habitat D exhibited a mixture of deposited tailings and intact native substrates while habitats E and F exhibited extensive deposited tailings and distinctions were made based on material texture (Habitat F exhibiting primarily fine grey tailings). Habitat types were associated closely with the four major water bodies identified in this study. Hazeltine Creek exhibited the greatest impact from the disturbance, with the depth of erosion of soil and parent material exceeding 7 m in some areas, exposing bedrock and depositing thick (~1-3m) tailings and associated sands in a corridor ranging from 40–250 meters wide.

The shoreline of Bootjack Lake consisted of mostly habitats A and B and lacked deposited tailings. The Lake exhibited extensive backwater depressions in its terrestrial shoreline that were organic rich, under-layed with an occlusive layer of highly reduced clay based on clear gleying and lack of oxidized rooting zones. As a result, Bootjack Lake sample layers were distinctly different in composition, where surficial samples were largely comprised of organic material, and with the exception of the outlet to Bootjack Creek, deep samples were composed of reduced clays. Bootjack Lake sediment deposits were primarily fine sediments imbedded in rocky cobbles. Hazeltine Creek, the main flow path of the released tailings, was scoured and almost entirely replaced by sediment deposited from the tailings outwash (habitat F). For both Polley and Quesnel Lakes, some sites close to the deposition path were replaced by tailings. Even though a large amount of tailings was pushed into Polley Lake at the south end [4], there were still some sites where natural diverse wetland communities were intact. The Quesnel Lake shorelines adjacent to the Hazeltine Creek delta where tailings entered the lake contained little vegetation and comprised mostly deep sand deposits with exposed weathered cobbles (habitats C & D). Only sites 20A and 21A on Quesnel Lake were vegetated, though only the most proximal sites to the Hazeltine outflow exhibited evidence of impacts from the breach (deposited tailings and/or displaced sediment). The markedly different physical features of the Quesnel Lake shoreline versus those in Bootjack Lake and at the northern end of Polley Lake reflect condition differences that existed before the spill. Quesnel Lake’s shoreline is characterized by extensive wave-washed cobbles and little vegetation within 10 m of the lake edge, even in sites sampled at greater distances from the Hazeltine Creek outflow (site 14A is 2.5 km from the Hazeltine Creek outflow and exhibited little vegetation cover and no evidence of deposited tailings).

Soil moisture and organic matter (SOM) contents of the samples collected as determined by oven drying and loss-on-ignition tests varied from 1.7% to 91.1%, and 0 to 854 g/Kg respectively, with SOM significantly associated with moisture content (R^2^ = 0.74, F = 401, P<0.001) (S1 Fig). Although there were no statistically significant differences in moisture content and SOM between layers and habitats, habitat A contained higher SOM than the other habitats, with habitat F samples having the lowest amounts of SOM (S2 Fig). When comparing SOM by lake/stream, Hazeltine Creek surficial samples contained significantly lower SOM relative to all Polley Lake sample layers (p_surf_ = 0.02, p_deep_ = 0.01, p_sed_ = 0.04) and Bootjack surficial samples (p = 0.02) based on Bonferroni-corrected Mann-Whitney pairwise comparisons (Kruskal-Wallis H = 46.15, p<0.0001)(S3 Fig). Deep samples collected from Hazeltine Creek contained significantly lower SOM than Polley Lake deep (p = 0.02) and surficial samples (p = 0.01). All other comparisons were not significant.

Pore-water chemistry properties were measured in lakeshore sub-aqueous sediments (S2 Table and S4 Fig). Temperature variations were due to the two different sampling periods, 6–8 and 21–23 October 2014, during which the average pore water temperatures were 13 and 9°C, respectively. Polley and Quesnel Lake sediments had similar circum-neutral pHs (7.3_±0.7_ and 7.8_±0.3_, respectively), whereas the pH in pore-water from Bootjack Lake was lower (average 6.5_±0.4_, p < 0.01) and Hazeltine Creek higher (average of 8.9_±0.3_, p < 0.001). The sediments within Quesnel Lake were the most aerated (DO = 7.7_±1.4_ mg/L) with those in Bootjack Lake having the lowest oxygen concentrations (DO = 2.2_±0.8_ mg/L, p < 0.001). A wide range of dissolved oxygen concentrations were measured in the Hazeltine Creek pore-waters. Conductivity was slightly higher in Polley Lake sediment pore-waters compared with Bootjack and Quesnel Lakes (p < 0.05). No conductivity data were obtained for Hazeltine Creek (sensor malfunction).

The ammonium-N, nitrate-N and phosphate concentrations in Bootjack Lake sediment pore water samples were all below 1 mg/L (S2 Table). There was more nitrate-N in the Quesnel Lake sediments (1.35_CI = 0–2.92_ mg/L) with the highest concentration found at site 12A (4.11 mg/L), which was a site where tailings were deposited. Nitrate-N concentrations were also higher in Hazeltine Creek than in the other locations (average of 2.34_CI = 0–5.75_ mg/L) with the highest concentration found at site 36A (8.24 mg/L).

In Hazeltine Creek, the average sulphate concentration of those sites from which pore water could be recovered was 251_CI = 0–258_ mg/L. One site had a sulphate concentration as high as 940 mg/L (site 36A)(S3 Table), which was also where the highest nitrate-N concentration was measured. At the Polley Lake sites, sulphate concentrations were higher than those in the other Lakes (Bootjack and Quesnel), but not as high in Hazeltine Creek (average of 125_±56_ mg/L). Sites with the highest sulphate concentrations were at locations containing tailings (sites 58A and 60A). However, one far site that was highly vegetated (site 53A) also had a high sulphate concentration. Sulphate concentrations were not measured in the pore waters sampled from Bootjack Lake and Quesnel Lake. Samples taken from Quesnel Lake since the impoundment failure found sulphate to be less than 15 mg/L [4]. Taken together soil and pore water characterization tests revealed that the deposited tailings created distinct habitats and environments compared with unaffected sites.

### Microbial composition

#### Diversity and distribution

**Fig 5:**
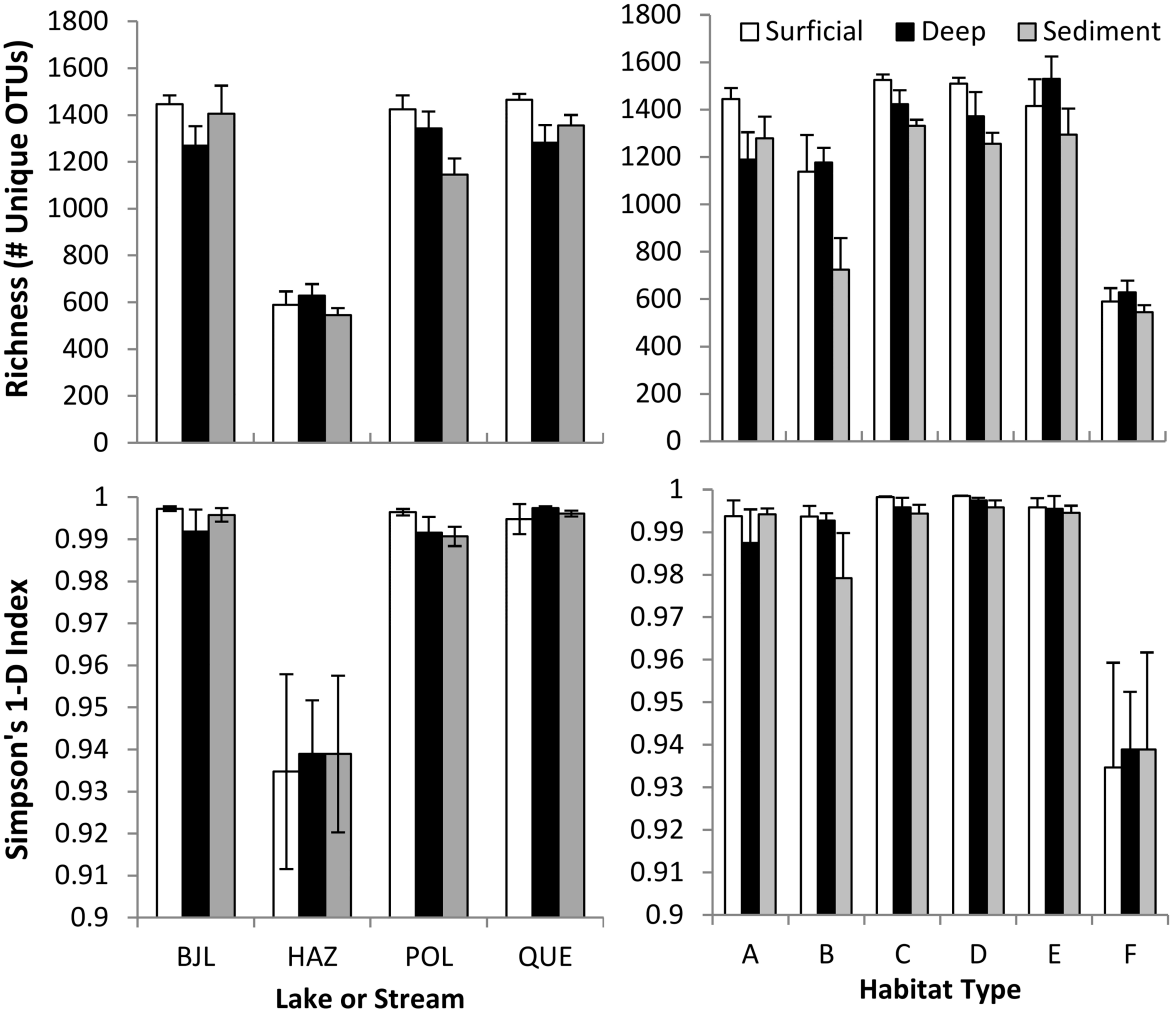
Species richness (sobs) and diversity (Simpson’s 1-D) of microbial communities in the surficial, deep and sediment layers in Bootjack Lake (BJL), Hazeltine Creek (HAZ), Polley Lake (POL) and Quesnel Lake (QUE). Comparisons are also shown for the six habitat types (A-F) identified in Fig 2. Error Bars represent standard error of the mean.

A total of 1,650,752 high quality sequences with a mean length of 227_±40_ bases were obtained from 234 samples with an average read count per sample of 5,630_±2,839_. After subsampling to 3,000 reads per sample, 15 samples were rejected due to insufficient sequencing depth. The impact of the tailings deposition was most apparent for Hazeltine Creek where species richness was approximately one third less than that observed for all the other sites. Although Hazeltine Creek was scoured and much of it filled with tailings, microorganisms were still detected in all layers of those sites that yielded enough DNA for sequencing. As expected, the un-impacted reference site, Bootjack Lake had the greatest species richness (observed number of species (sobs) in Fig 5), especially in the surficial layers though it only differed significantly from Hazeltine Creek (F_3,21Sed_ = 9.3, F_3,29Surf_ = 49.0, F_3,26Deep_ = 17, p<0.0005). As pre-event microbial community data are not available, the heightened diversity in Bootjack Lake may be the result of pre-existing differences among water bodies as Bootjack exhibited higher soil organic matter reflective of elevated productivity.

Comparisons of species richness and diversity revealed a significant 57% reduction in richness for Hazeltine Creek relative to the remaining sites (F_3,21Sed_ = 9.3, F_3,29Surf_ = 48.8, F_3,26Deep_ = 17, p<0.0005) and a concomitant decline in diversity based on Simpson’s 1-D (F_3,21Sed_ = 16.6, F_3,29Surf_ = 6.0, F_3,26Deep_ = 10.4, p<0.005). These patterns were reflected in similar declines in the most highly impacted sites (Habitat F), as the majority of these sites were collected within the Hazeltine Creek corridor. In addition to having fewer species in Hazeltine Creek, these comprised mostly of a few very dominant ones (explained in greater detail in the following *Indicator Species Analysis* section). This pattern did not appear to be homogeneous across the site, especially in the deep layer (25–30 cm below the surface). In the surface layers of Hazeltine Creek, microbes were present in some sites concentrated in a reduced number of species.

Polley Lake showed the greatest intra-lake differences in species richness where some sites had far fewer species than average, especially in samples taken from the deep mineral layers and sub-aqueous sediments. It appears as if there might be several distinct microbiomes in Polley Lake, owing to its wide within-lake variation in diversity. Sites with low species richness were located at either end of the lake and did not appear to be correlated with presence of tailings (S5 Fig). This may be interpreted in light of classic disturbance theory where an intermediate position along the disturbance gradient would be expected to yield increases in alpha diversity [41]. This theoretical framework also provides an explanation for the observed pattern in beta diversity (discussed below) as Polley Lake encompasses the widest spectrum of disturbance pulse intensities which would support distinctly different microbes [42].

Site diversity was also compared with respect to the habitat types (Fig 5). Habitat F exhibited significantly lower observed richness (# of distinct OTUs) and diversity (Simpsons 1-D) for surficial (H_OTU#_ = 24.15, p = 0.0002, H_Simpson’s 1-D_ = 23.62, p = 0.0002) and deep (H_OTU#_ = 22.15, p = 0.0004, H_Simpson’s 1-D_ = 21.66, p = 0.0006) samples. For sediment, habitat F differed significantly from habitats A and B, while habitat B differed significantly from C (H_OTU#_ = 14.22, p = 0.014, H_Simpson’s 1-D_ = 13.36, p = 0.02). Non-parametric Kruskal-Wallis test (H) and associated Mann-Whitney pairwise comparisons are reported wherever a preliminary Levene’s test indicated significant heteroscedasticity. The heterogeneity (and reduced mean richness) observed in habitat B likely reflects the substrate heterogeneity of Polley Lake where these samples were collected. It is worth noting that habitat B samples occurred in backwater depressions that were adjacent to the breach impact area. The heterogeneity (and reduced mean richness) observed in habitat B likely reflects the substrate heterogeneity of Polley Lake where these samples were collected.

#### Compositional comparisons

**Fig 6:**
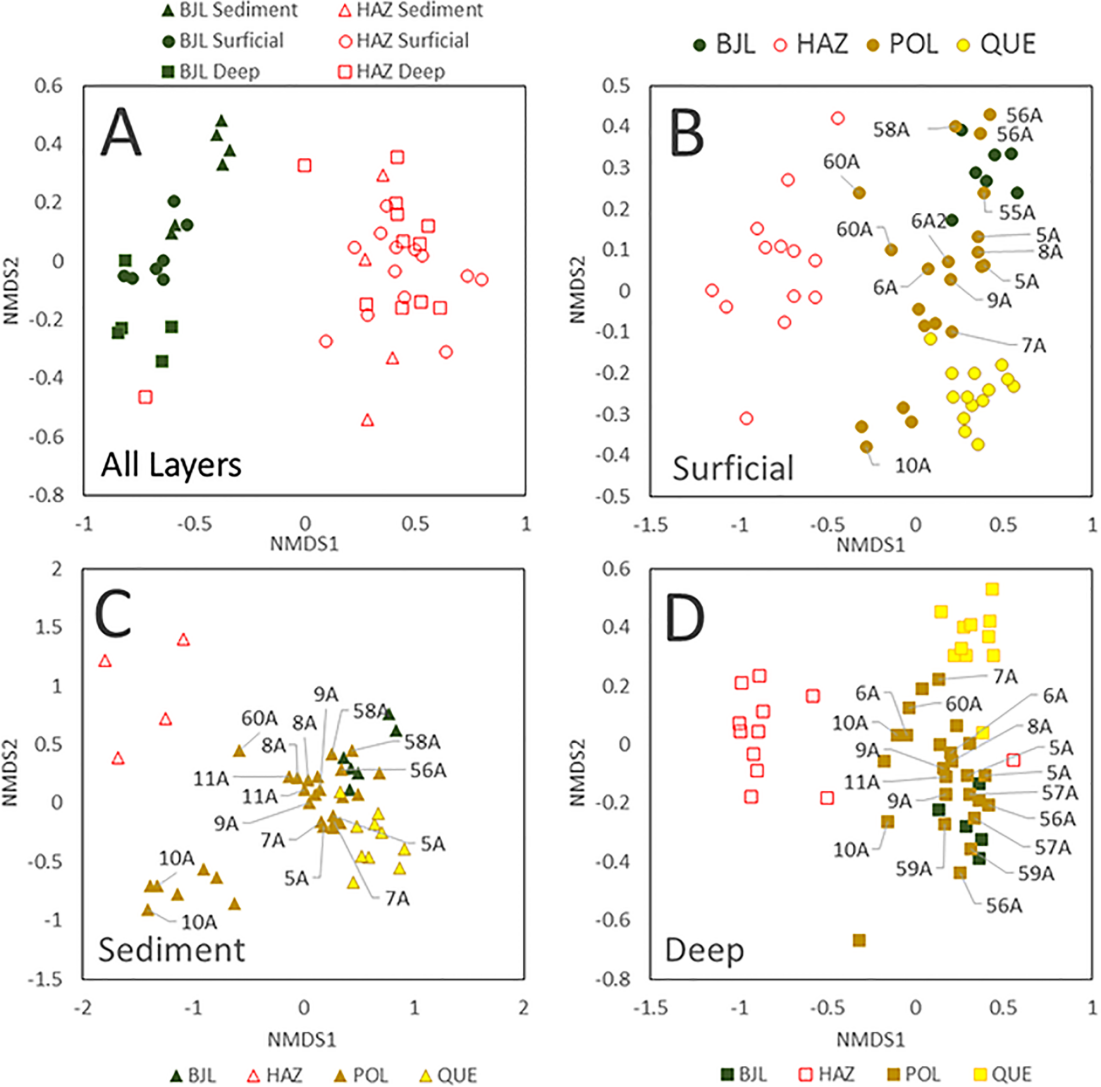
Ordination plots comparing. (A) the microbial population structures of sites in Bootjack Lake (BJL) and Hazeltine Creek (HAZ) (symbol shapes reflect layers from which samples were collected), (B) the surface layer microbial population structures among the lakes and Hazeltine Creek, (C) the deep layer microbial population structures from the different lakes and Hazeltine Creek, and (D) the sediment layer microbial population structures from the lakes and Hazeltine Creek. Sites on Polley Lake (brown symbols) closest to the impoundment are labeled with their site numbers.

Major site differences were reflected in the composition of microbial communities at the landscape scale. Graphs in Fig 6A–6D represent whole community dissimilarity contracted to two axes of variation. In these plots, distance between points (sites) represents approximate community dissimilarity.

The two sample replicates taken for each site are included as independent points in order to evaluate the degree of site-specific consistency in microbial community composition. These communities are clearly sacrificial pseudoreplicates, and site community means were used wherever inferential statistics were applied to compare sites.

First, the microbial community compositions observed in the most disturbed landscape, Hazeltine Creek, were contrasted with those found within the reference site, Bootjack Lake (Fig 6A). There are general differences in the microbial composition of lentic versus lotic systems in the absence of disturbance [43,44], so it would be improper to expect Hazeltine Creek to have a great degree of similarity to Bootjack Lake even before the disturbance. The literature is lacking in the degree to which this difference might be anticipated in magnitude or direction except to say that connected systems tend to share microbial communities (a property that attenuates with distance) [43] and similar systems within biomes share microbial qualities relative to different biomes [45]. As a result, it is not surprising that there is near complete segregation of Hazeltine Creek communities from those in Bootjack Lake for all layers. Additionally, there was a delineation of microbial communities by layer in Bootjack Lake, which was not seen in Hazeltine Creek. This may be a property of shallow lotic systems, but the lack of segregation by layer found in Hazeltine Creek is consistent with the physical mixing of layers caused by tailings flow and scouring.

An additional structural difference between the Bootjack Lake and Hazeltine Creek microbial communities was the dissimilarity between sites. Compositional variations of the layer microbial ecological niches in Bootjack Lake were more constrained across sites than was the case for Hazeltine Creek where there were greater dissimilarities between sites. It was observed for both Bootjack Lake and Hazeltine Creek that microbial community structures across layers from the same site were no more similar to each other than to samples from other sites.

Since the reference site exhibited stratification of microbial habitats by layer, the population structures of all samples taken from all lakes and Hazeltine Creek were contrasted for each layer microbiome. In all layers, the microbial populations within the disturbed area of Hazeltine Creek were compositionally distinct from those found in the lake samples. This is anticipated given inherent differences in lentic versus lotic systems, but the extent of impact experienced by this disturbance would suggest an even greater degree of segregation than might be explained by pre-disturbance differences. Sites found along Hazeltine Creek with the greatest compositional similarity to Polley Lake (sites 43A and 41A) were positioned at the midpoint of the stream and near Quesnel Lake, rather than immediate to the Polley Lake outlet (Fig 1). Additionally, segregation of Quesnel Lake communities from the remainder of the study sites was observed also for all layers. These systems are unique, bearing markedly different substrates in terms of both mineral composition and organic content. Hazeltine Creek was dominated by sediment deposited from the tailings outwash within the studied depths and Quesnel Lake exhibited cobbled/sandy surficial layers over deep sandy deposits. Although Quesnel Lake sites close to Hazeltine Creek delta within the disturbed area had mineral or mixed tailings habitats, their microbial community structures were not similar to those observed in the main deposition zone of Hazeltine Creek suggesting that the disturbance had little impact on the near shore microbiomes of Quesnel Lake. Bootjack Lake habitats contrasted with these aforementioned sites since they were highly vegetated, which contributed to its dissimilar population structure. Polley Lake surficial microbiomes varied widely according to site and shared structural characteristics with some sites in Bootjack Lake, Quesnel Lake and Hazeltine Creek. The labeled sites in Fig 6B–6D (58-60A) were associated with a contiguous emergent marsh community that was buried in tailings at its southern extent (site 60A) and exhibited infiltration and deposition of fine tailings at its northern extent (58A). The intervening marsh community represented by 59A remained intact with little macroscopic indication of tailings deposition. Both replicate samples taken from site 60A contained microbial community structures that resembled those found within Hazeltine Creek. In contrast, sites 58A and 59A had a microbial composition more similar to those found in the highly vegetated Bootjack Lake terrestrial surficial layers. Few studies have evaluated the role microbial community composition and alpha/beta diversity play in resilience to disturbance. This high level of within-lake beta diversity may afford the microbiota of Polley Lake a degree of resilience to long-term impacts from the breach (especially in comparison with Hazeltine Creek) by providing reservoirs of metabolic potential.

In the deeper terrestrial layers, with one exception, microbiomes within Hazeltine Creek were structurally distinct from those around the lakes (Fig 6C). In this layer, many sites from Polley Lake and Bootjack Lake were similar to each other. Quesnel Lake microbiomes retained their distinct structure. The effect of deposited tailings on Polley Lake microbiomes was mixed with some sites near the tailings deposition resembling Hazeltine Creek communities and others retaining characteristics of the further sites on Polley Lake and Bootjack Lake microbial populations. Similar observations were made for the sub-aqueous sediment microbiomes where most lake sites exhibited microbial population structures that were distinct from the Hazeltine Creek microbiomes (Fig 6D). A pairwise comparison of the sediment microbial population structures in Bootjack Lake, Polley Lake, Quesnel Lake and Hazeltine Creek using the analysis of molecular variance (AMOVA) method [46] revealed that the microbial population structures were significantly dissimilar (p < 0.001). To further explore how these microbiomes differed from each other, an indicator species analysis was performed to identify taxa that were statistically more prevalent in each of the hydrozone layers in each lake or Hazeltine Creek.

#### Indicator species analysis (all sites)

An indicator species analysis identifies species that exhibit consistency in presence and prevalence associated with a defined treatment or condition—i.e. identified species are distinct relative to the remaining treatments or conditions. The indicator species analysis conducted here revealed that 24–28% of terrestrial species (OTUs) in Hazeltine Creek were higher (p < 0.001) in relative abundance in the Creek than in the other sites (Table 1). These accounted for 60% of the relative abundance of all microbial taxa in Hazeltine Creek.

**Table 1:** Number of indicator species identified for Polley Lake, Bootjack Lake, Quesnel Lake and Hazeltine Creek within each hydrozone layer.

Lake	# of Sites	# of OTUs (total)	Indicator sp. (#)	Representation (% of OTUs)	Rel. abund. (% of reads)
*Terrestrial Surface Layers*					
Polley Lake	16	1663	32	1.92	3.4
Bootjack Lake	14	1546	182	11.77	31.1
Quesnel Lake	16	1423	220	15.46	26
Hazeltine Creek	21	745	211	28.32	61.3
*Deeper Terrestrial Mineral Layers*					
Polley Lake	25	1663	13	0.78	1.4
Bootjack Lake	16	1546	158	10.22	39.2
Quesnel Lake	11	1423	235	16.51	41.2
Hazeltine Creek	16	745	179	24.03	59.6
*Sub-aqueous Sediment Layers*					
Polley Lake	26	1663	49	2.95	9.69
Bootjack Lake	19	1546	179	11.58	36.85
Quesnel Lake	12	1423	137	9.63	33.41
Hazeltine Creek	7	745	75	10.07	47.67

**Fig 7:**
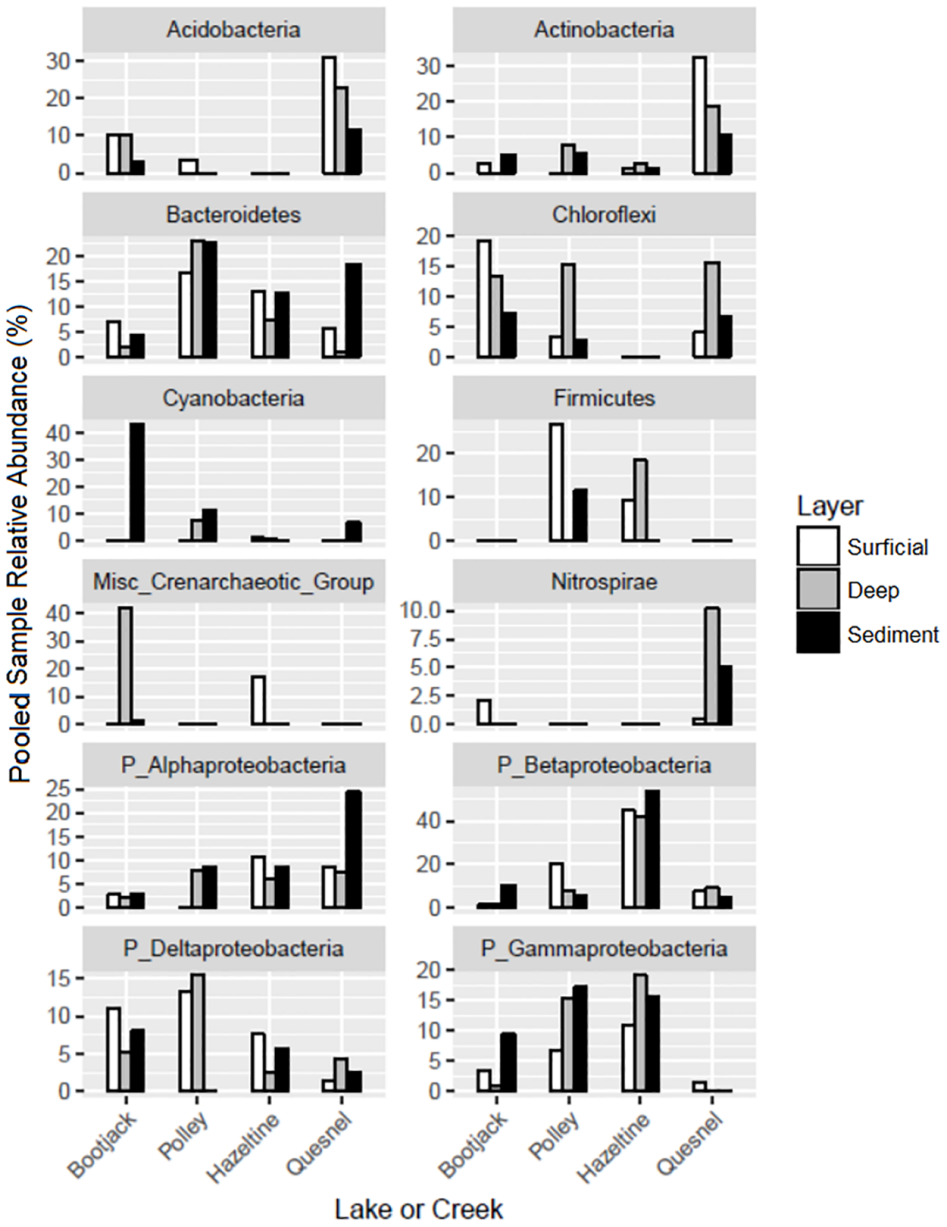
An indicator species analysis was conducted to further facilitate exploration of the microbial community database. Bars represent percent contribution to total recovered DNA that was attributable to each of a number of phyla that maximize contrasts amongst the four study systems.

Many of these indicator species (42–47%) were classified as Betaproteobacteria (Fig 7). Betaproteobacteria orders found throughout the lakes and Hazeltine Creek were Burkholderiales, Hydrogenophilales, Rhodocyclales and Methylophilales. The Betaproteobacteria indicator species in Hazeltine Creek were members of the Hydrogenophilales order, specifically the genus *Thiobacillus*. Betaproteobacteria were also found in the Bootjack, Polley and Quesnel Lake sites, but these were classified in the other Betaproteobacteria orders. Members of the Burkholderiales are known for their capacity for bioremediation of metals and hydrocarbons [47], and Rhodocyclales are reported to be involved in denitrification, selenate reduction, and dechlorination [48]. Gammaproteobacteria were less abundant than alpha and betaproteobacteria across sites, predominated by *Pseudomonas* sp. and members of the order Xanthomonadales. Gammaproteobacteria are diverse functionally, and often associated with terrestrial sources [49](e.g. plant root-associated *Pseudomonas* species) and the lack of abundance of Gammaproteobacteria on the Quesnel Lake shoreline could be attributed to a number of factors including the lack of developed soil and vegetation structure, distance and connectivity to sources within the surrounding forest, or dilution effects resulting from the extreme differences in lake surface areas.

Quesnel Lake microbiomes were distinguished from the other sites by the relative abundance of approximately 9–15% species that were different taxonomically depending on the layer. In all layers, these included species classified in the Acidobacteria and Actinobacteria phyla. In the deep layers, *Nitrospira* spp. in the phylum Nitrospirae were also indicator species and, in the subaqueous sediments, indicator species classified in the phyla Alphaproteobacteria and Bacteriodetes were also identified. Actinobacteria are widely distributed soil and sediment microorganisms that perform a versatile range of ecosystem functions such as nitrogen fixation and organic matter decomposition [50]. Acidobacteria are similarly ubiquitous in aquatic soils and sediments and some species are notable for their tolerance of mildly acid environments and psychrotolerance [51]. *Nitrospira* contribute to nitrification by performing nitrite oxidation to nitrate [52]. The Bacteroidetes phylum Quesnel Lake aqueous sediment indicator species were classified in a family of species known for degradation of complex organic matter (Chitinophagaceae) [53]. Alphaproteobacteria indicator species in Quesnel Lake sediments were classified in orders Rhizobiales, Rhodobacterales and Sphingomonadales, which contain metabolically flexible species capable of existing under aerobic as well as anaerobic conditions, fixing C and N, and that have been noted as drivers for bioremediation of contaminated soils [54] [55]. Taken together, the indicator species of Quesnel Lake portray a diverse community of species carrying out many essential ecosystem services that were minimally impacted by the tailings dispersion.

Overall, Bootjack Lake and Polley Lake had more overlap in microbial community composition in all layers (Fig 6). Bootjack Lake exhibits extensive backwater depressions in its terrestrial shoreline that are organic-rich, underlayed with an occlusive layer of highly reduced clay (based on clear gleying and lack of oxidized rooting zones). This vertical segregation of substrates and presumably very different biogeochemical conditions leads to both segregated microbial communities and differences in community composition relative to the inorganic substrates found in Hazeltine Creek and Quesnel Lake. Bootjack Lake lacks direct deposition of tailings material in the surficial layers and lake sediments. Absence of any recent documented disturbance in Bootjack Lake precluded mixing of the vertical layers and their microbial communities, thus the indicator species taxa particular to Bootjack Lake layers reflect niche partitioning that is characteristic of stable undisturbed sites. For instance, most (71.3%) indicator species Chloroflexi that were higher in relative abundance in the Bootjack Lake terrestrial organic-rich surficial layers were classified in the Anaerolineaceae family, which contains species associated with anaerobic digestion and fermentation of organic matter that are possibly syntrophic with methanogens [56]. The Miscellaneous Crenarchaeotic Group in the deeper terrestrial layers of Bootjack Lake are Archaea about which little is known as these species are difficult to grow outside of their natural habitat. They are thought to contribute to organic matter cycling, are noted for being ecologically important in both fresh and saline environments, and their specific functional roles are yet to be fully explored [57]. Cyanobacteria in the sub-aqueous sediment layers likely settled there from the water surface after blooms occurred, which are common during warmer temperatures and when lake nutrient levels are high.

Unlike the other lakes and Hazeltine Creek, Polley Lake microbiomes were distinguished by few indicator species, which comprised only approximately 1–3% of the total number of OTUs in Polley Lake (Table 1). There appeared to be two distinct sub-aqueous sediment microbial population structures in Polley Lake. These were not correlated to the impact of the disturbance, and more likely were due to the diversity of habitats seen along the Polley Lake shoreline. In contrast to Bootjack Lake, Polley Lake had an intermediate level of stratification as there were few differences in indicator species for the Polley Lake hydrozones and they were classified over a wider variety of Phyla (dominated primarily by taxa within all Proteobacteria classes, as well as Bacteriodetes and Firmicutes phyla). As Polley Lake was the closest water body to the tailings impoundment dam breach location and since the habitats within Polley Lake were diverse, microbial compositional variations within Polley Lake were explored in more detail.

#### Taxonomic comparison within Polley Lake

**Fig 8:**
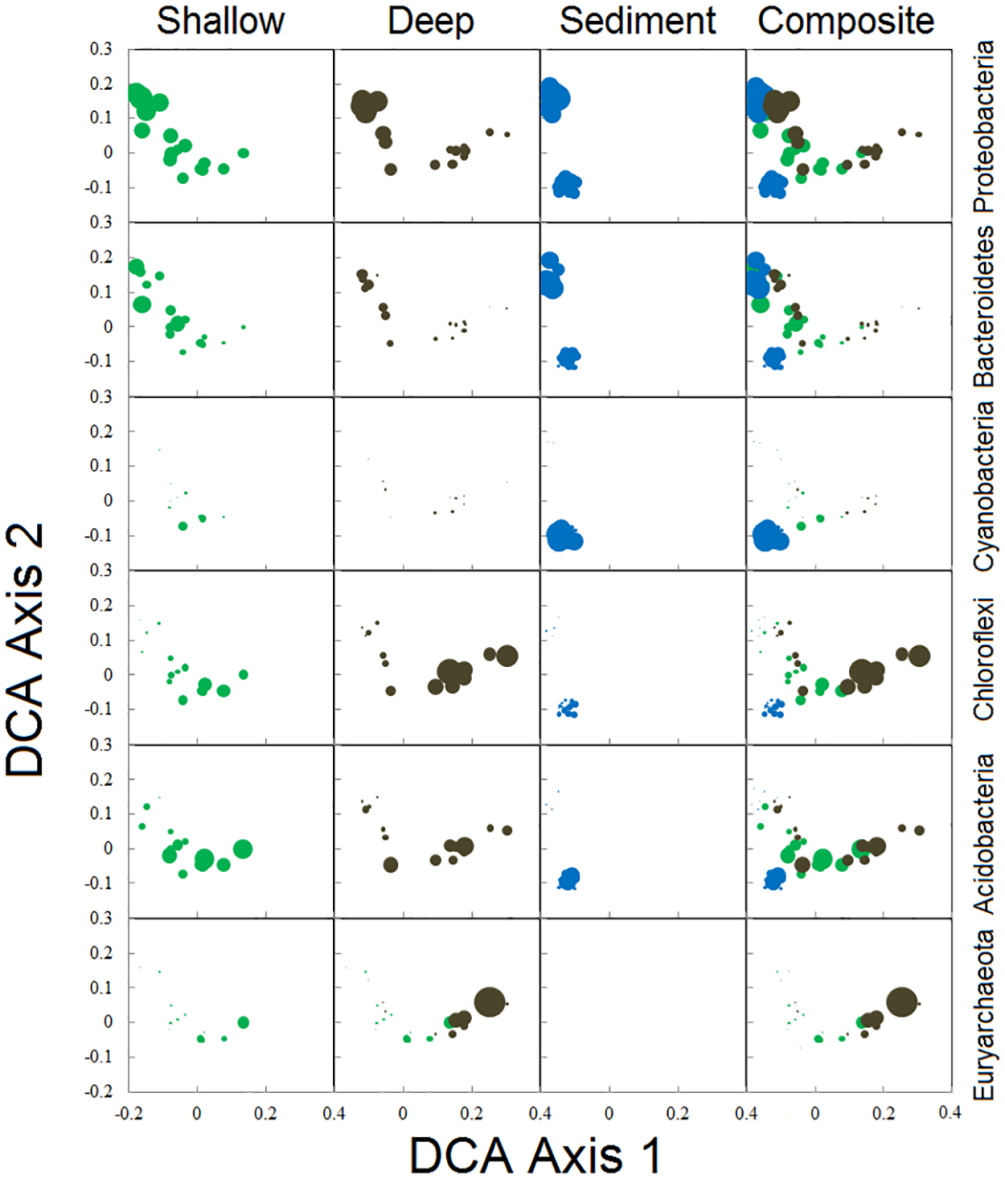
Detrended correspondence analysis depicting Polley Lake community dissimilarity based on reconstructed OTUs for each of the three sample types. Bubble size varies in proportion to relative abundance of each major taxonomic group (Phylum) listed to the right. Bubble color highlights the three sample types collected (green = surficial, black = deep, and blue = sediment).

Polley Lake was of particular interest, as it reflected a spectrum of impacts from the impoundment failure, and examples of microbial communities exhibited similarities to the remaining sample systems. When representing taxonomic variation in the form of relative abundance of the dominant Phyla (Fig 8), some clear patterns emerge.

At the highest relative abundance, Proteobacteria and Bacteriodetes are most dominant at the northern end of Polley Lake. These groups are quite diverse metabolically and tend to increase in abundance in areas with high levels of carbon mineralization [58]. This is consistent with our observations, where deep peat deposits coincide with increases in these groups. Cyanobacteria are associated with sediments found at the southern end of Polley Lake. Cyanobacteria are photosynthetic and effectively absent from terrestrial sample cores. MPMC staff reported an algal bloom in Polley Lake prior to the failure (pers. comm.), possibly accentuating this effect. This is in contrast to the northern sites where cyanobacteria abundance is relatively low and suspended algae are prevalent. Although Chloroflexi are known to associate in a spatially segregated manner with cyanobacteria in thermal environments [59], there is only a weak association between these groups in Polley Lake sediments, suggesting this relationship is environmentally constrained. Chloroflexi abundance peaks in deep terrestrial substrates at the south end of Polley Lake, possibly indicating a relative increase in sulfide in these areas, which some chloroflexi representatives use as an electron donor [60]. Based on a qualitative comparison (Fig 8), Acidobacteria follow a similar pattern as chloroflexi for deeper samples, but appear to increase in abundance in all substrate types near the impoundment failure origin. The most prevalent taxa identified as Acidobacteria were unclassified beyond the phylum level in uncultured environmental groups (iii1-15 & RB41). The common Acidobacteria for which order-level classification was obtained included members of the orders Solibacterales, known to produce biofilms that reduce accessibility of substrates to sulfide oxidation by chemolithotrophs [61], Acidobacteriales, and Holophagales. A post-hoc analysis of Acidobacteria abundance comparing visibly impacted sites (6A & 57A-60A) on the south end of Polley Lake to those that were not apparently impacted (7A-11A) failed to detect significant differences in Acidobacteria abundance both within and among sample layers (F_5,23_ = 1.82 p = 0.15). Finally, members of the Euryarchaeota are prevalent at the south end of Polley Lake, the most abundant of which are members of the orders Methanosarcinales (Families Methanosaetaceae and Methanosarcinaceae) and Methanomicrobiales (Family Methanobacteriaceae) that are obligate anaerobes restricted to deeper sediments. This was expected for undisturbed deep sediments as members of these orders are methanogens that typically are found in highly reducing, anoxic environments.

#### Thiobacillus

Of the Betaproteobactera indicator species prevalent in the Hazeltine Creek deposition zone, 52% were classified in the genus of *Thiobacillus* (Order Hydrogenophilales). One particular OTU classified as *Thiobacillus*, OTU1, can be considered a biotic indicator for tailings deposition and metabolism outside of the tailings impoundment as its relative abundance clearly coincides with the most disturbed areas, which were in the main flow path of the breached material along Hazeltine Creek (S6 Fig). Porewater pH measurements within the tailings deposition zone were basic rather than acidic [62]. These results are consistent with mineralogical assessments of the deposited material made by SRK consulting indicating a low potential for acidic rock drainage formation [40]. *Thiobacillus*-related OTU1 appears to be a native microorganism since it was identified at most other sites and hydrozones including in the reference site of Bootjack Lake (S6 Fig). Further phylogenetic classification of the *Thiobacillus* OTU1 revealed that it is closely related to species of the *Thiobacillus thiophilus*, *T*. *denitrificans*, *T*. *thioparus* clade (Kellermann 2009). Species in this clade are chemolithoautotrophs, which means they do not need organic carbon compounds for growth since they utilize CO_2_ instead. Thus they thrive in the organic matter deprived, mainly mineral environment, of the main deposition zone over heterotrophs that require organic carbon. These *Thiobacillus* species are involved in oxidation of intermediate redox sulfur compounds and are facultative anaerobes that are capable of denitrification in the absence of oxygen [63]. High relative abundance of *Thiobacillus*-related OTU1 within all layers in Hazeltine Creek might be explained by the presence of nitrate within the pore water of the tailings material. Nitrate is a chemical component of explosives used in mining and is often not completely consumed in the explosive reaction leaving residual amounts in the tailings. It is not known if *Thiobacillus* colonized the deposited material from surrounding natural areas or was already growing in the tailings and was transported with the spilt tailings into the deposition area. Its oxidation of sulfur compounds might have contributed to elevated sulfate concentrations within the deposited material. However, the tailings pore water sulfate concentrations were around 500 mg/L when the tailings material was still within the storage impoundment and measured sulfate concentrations within Hazeltine Creek, where it was possible to determine these, were mostly less than 500 mg/L except for one location. The denitrification ability of the *Thiobacillus thiophilus* clade suggests that *Thiobacillus*-related OTU1 in the disturbed zone potentially is capable of stemming the release of nitrate from tailings substrates [63,64] and may contribute to passive remediation of nitrate within the affected watershed.

#### Metabolic potential of microbial communities

**Fig 9:**
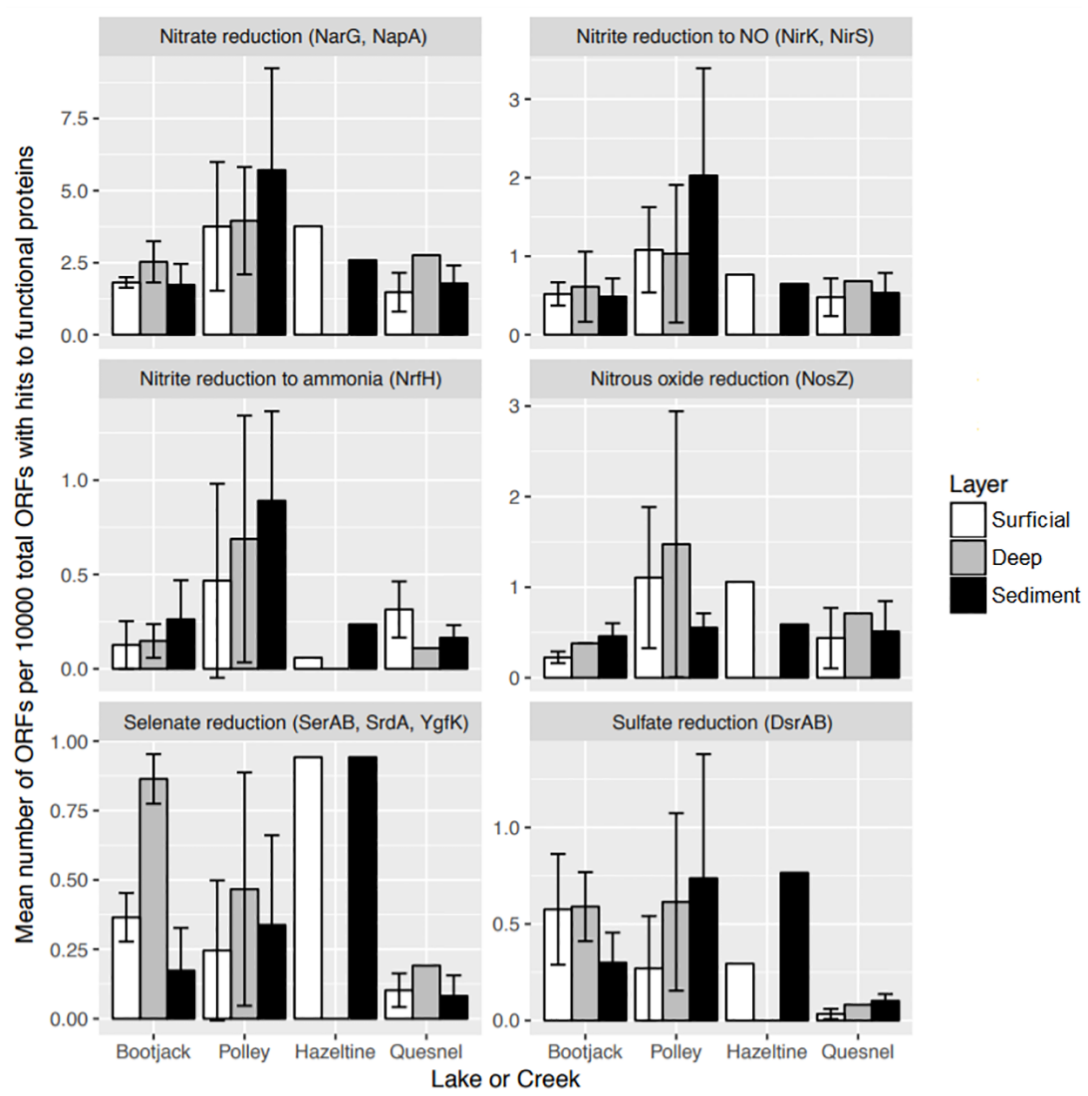
Protein sequence read frequencies within the metagenomic record. The data here represent open reading frames within the recovered genomic DNA that code for enzymes involved in the listed reactions. The following enzymes are involved in the reduction of nitrate (NO_3_^-^) and intermediate compounds to Ammonia (NH_3_) (NarG, NapA, NirK, NirS, NrfH, & NosZ) via denitrification.

An important question after a catastrophic event such as a spill of chemically distinct material into the natural environment is how the ecosystem might respond metabolically. We found structural differences in the distribution and types of microorganisms in the disturbed areas and to a certain extent what is known about closely related cultured species could hint at the metabolic properties of the community. But many of the microorganisms in these sites could not be identified to the Genus level and nothing is known about their capabilities. Sixty samples were subjected to whole genome sequencing providing an average of 145 million bases of sequence per sample and an average of 162,691 open reading frames per sample after quality control filtering. Given that the communities sequenced contained well over a thousand observations, we did not expect this level of sequencing depth to cover complete genomes of all members of the community. Nevertheless the sub-sample of genomic material provides partial insight into the metabolic potential of presumably the most dominant community members. An arbitrary cut-off of 30 million base pairs per sample eliminated 13 samples from further analysis. For functional annotation, enzymes for the dissimilatory denitrification, selenate reduction and dissimilatory sulfate reduction pathways were chosen since nitrate, selenate and sulphate are chemical compounds known to be present at concentrations above background in the tailings pore water (personal communication with Mount Polley Mine personnel). For the analyzed samples, the number of ORFs with hits to each of the selected target proteins was averaged for each hydrozone in all sites within each Lake or Hazeltine Creek (Fig 9).

Representation of the denitrification pathway enzymes within the hydrozones of Polley Lake was highly variable across sites, which reflects the wide species compositional differences observed for the Polley Lake communities. Higher proportions of denitrification enzymes were found in Polley Lake versus any of the other sites, although all sites displayed the potential for denitrification both to dinitrogen gas and ammonia. The metabolic potential for selenate reduction via putative selenate reductases SerAB, SrdA and YgfK was detected for all sites and hydrozones especially for the two samples from Hazeltine Creek that yielded sufficient DNA for metagenomic analysis. The known selenate reductase SerAB belongs to *Thauera selenatis*, which uses selenate as its preferred electron acceptor [65]. Several OTUs from the compositional analysis were classified in the *Thauera* genus, although not enough information was available to identify them to the species level. The putative selenate reductase SrdA comes from *Bacillus selenatarsenstis*, a selenate and arsenate reducing bacterium [66]. Many OTUs were classified in the genus *Bacillus* as this is a broad genus containing many species. The putative selenate reductase YgfK is present in many different organisms since it is involved in selenium metabolism and selenium is an essential nutrient for synthesis of selenoproteins. All types of selenate reductases were potentially present in the Hazeltine Creek samples and the other sites displaying the metabolic potential for selenate reduction. The potential for sulfate reduction was present in all sites. There were noticeably fewer hits to selenate reductases and the DsrAB proteins for ORFs from Quesnel Lake. We hypothesize that this was due to the absence of highly vegetated and potentially anaerobic habitats, preferred by selenate and sulfate reducing bacteria, on the shoreline of Quesnel Lake. Sulfate reduction by sulfate reducing bacteria is an important mechanism for bioremediation of metal-containing mine-impacted water. The product sulfide reacts with metal cations to form sparingly soluble metal sulfides [67]. This has been confirmed as a metal removal mechanism in many constructed wetlands [68] [69]. The phylogenetic analysis revealed many OTUs classified as Deltaproteobacteria, which includes most sulfate reducing bacteria. Additionally two sulfate reducing bacteria genera, *Desulfosporosinus* and *Dehalobacter*, which are in the Clostridiales Order were also identified in many sites, especially those in Bootjack and Polley Lake (Fig 7), but also within Hazeltine Creek. The tailings contained low amounts of copper minerals, only a small portion of which were sulfides [40]. Although the deposited material was found to be resistant to leaching, the presence of the potential for sulfate reduction within the disturbed areas might prove to be a useful mechanism for metal sequestration should any metals leach from the deposited material in the future.

## Conclusions

Upon sampling within two months of the tailings impoundment failure, physical, chemical, and microbial properties differed between sites, reflecting pre-existing variations in environmental conditions and influences from the tailings released on site. As is often the case with major disturbance studies, ambiguity persists as to the relative importance of pre-existing site differences in driving the observed differences among sites. The sites with extensive tailings deposition (primarily Hazeltine Creek) exhibited an elevated pH and increases in sulphate and nitrate. Most remarkably, and despite a major influx of water and tailings material into Polley Lake, only the most proximal site (60A) exhibited microbial community characteristics consistent with those of the primary impact zone. In areas where tailings material were deposited over native organic-rich substrates (sites 58A & 60A), there was a surprising level of preservation of the microbial community composition at depth, suggesting a degree of resilience of community structure in subsurface peat deposits to changes in the surface environment.

Finally, the prevalence of specific taxa provides a window into the current and future metabolic properties of the tailings deposits. The prevalence of taxa associated with sulphate oxidation and denitrification. suggests a potential for passive remediation within the affected watershed. Chemically, the increase in soluble nitrate is of potential concern to wildlife in Quesnel Lake, however, the prevalence of *Thiobacillus denitrificans* and members of the order Rhodocyclales suggest that the microbiota may serve to attenuate the release of nitrate.

Mount Polley presents a prime opportunity to assess microbial community trajectories following disturbance, and resilience to further perturbations. Field experimentation is underway to evaluate the potential for biostimulation and biomagnification to promote beneficial microbial activity in the deposited tailings.

## Supporting information

S1 Fig Correlation of soil moisture and organic matter contents. SOM–soil organic matter. Linear (green) and 2nd order polynomial (red) contours are included.
(PDF)

S2 Fig Boxplots of SOM (g/kg) contents measures at all sites compared across layers and habitats. (PDF)

S3 Fig Soil organic matter (g/kg) content of samples collected from all three layers depicted according to geographical location. Point size reflects Soil organic matter content (g/kg).
(PDF)

S4 Fig Pore water pH, dissolved oxygen (mg/L) and conductivity (μS/cm). Box-whisker plots reflect medians, quartiles and outliers by lake/stream.
(PDF)

S5 Fig Species richness (sobs) in Polley Lake microbiomes. Point size reflects relative species richness (estimated as number of distinct OTUs).
(PDF)

S6 Fig Landscape-scale distribution of OTU1. Relative abundance (% of total bacterial read counts) is expressed for the three sample types.
(PDF)

S1 Table Transect origination coordinates are provided below for the 60 sites (in decimal degree format) with date, local (Pacific Standard) time of day (TOD) and site ID codes for the biomonitoring network. (PDF)

S2 Table Water quality metric means ± standard error. Fields in grey marked with (—) yielded insufficient data for reliable comparisons (<4 observations).
(PDF)

S3 Table Chemistry of the sediment layer pore water. Major water bodies are labelled as follows: BJL = Bootjack Lake, HAZ = Hazeltine Creek, POL = Polley Lake, QUE = Quesnel Lake.
(PDF)
