# Supplementary material for: Short-term microbial effects of a large-scale mine-tailing storage facility collapse on the local natural environment
Source: PLoS One. 2018 Apr 25;13(4):e0196032. doi: 10.1371/journal.pone.0196032 (PMC5918821; doi:10.1371/journal.pone.0196032)
Supplement: S1 Fig — SOM–soil organic matter. Linear (green) and 2nd order polynomial (red) contours are included. (PDF) [file pone.0196032.s001.pdf]

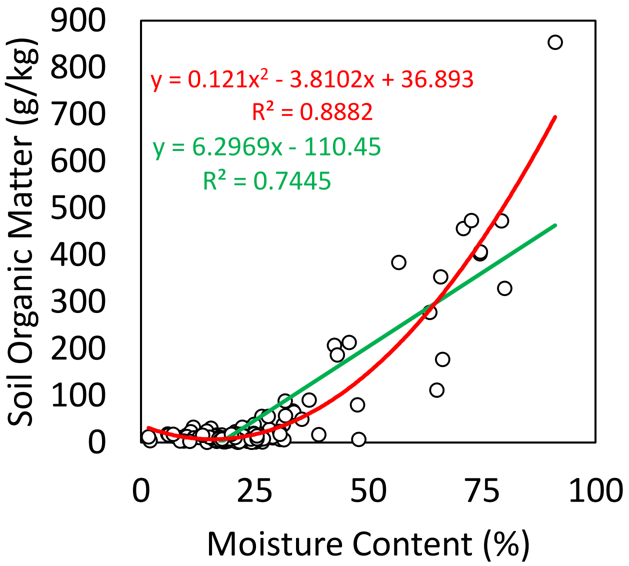

S1 Figure. **Correlation of soil moisture and organic matter contents**. SOM – soil organic matter. Linear (green) and 2nd order polynomial (red) contours are included.
