# Supplementary material for: Short-term microbial effects of a large-scale mine-tailing storage facility collapse on the local natural environment
Source: PLoS One. 2018 Apr 25;13(4):e0196032. doi: 10.1371/journal.pone.0196032 (PMC5918821; doi:10.1371/journal.pone.0196032)
Supplement: S2 Fig — (PDF) [file pone.0196032.s002.pdf]

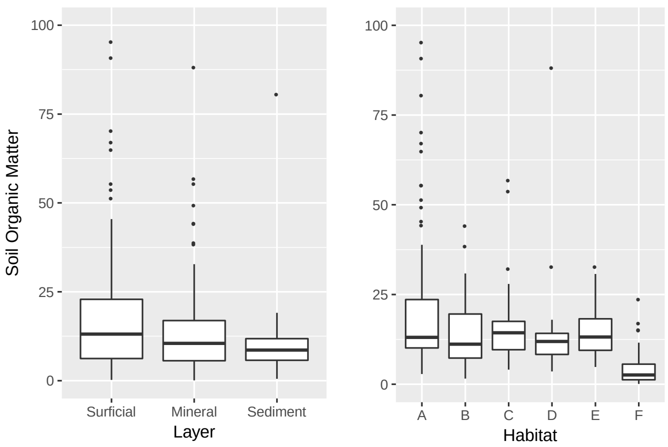

S2 Figure. **Boxplots of SOM (g/kg) contents measures at all sites compared across layers and habitats**.
