# Supplementary material for: Short-term microbial effects of a large-scale mine-tailing storage facility collapse on the local natural environment
Source: PLoS One. 2018 Apr 25;13(4):e0196032. doi: 10.1371/journal.pone.0196032 (PMC5918821; doi:10.1371/journal.pone.0196032)
Supplement: S3 Fig — Point size reflects Soil organic matter content (g/kg). (PDF) [file pone.0196032.s003.pdf]

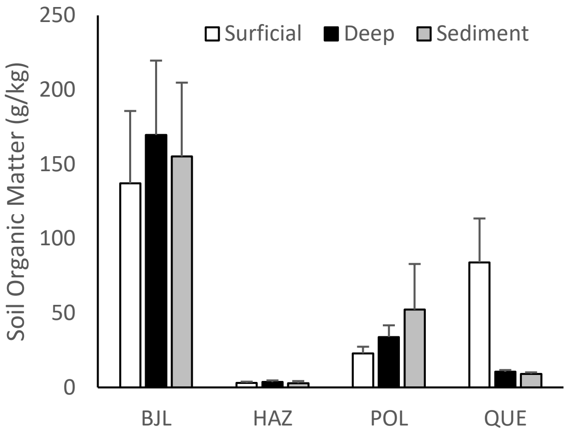

S3 Figure. **Soil Organic Matter (g/kg) comparisons by lake/stream.** Error bars reflect 2X Standard Error.
