# Supplementary material for: Short-term microbial effects of a large-scale mine-tailing storage facility collapse on the local natural environment
Source: PLoS One. 2018 Apr 25;13(4):e0196032. doi: 10.1371/journal.pone.0196032 (PMC5918821; doi:10.1371/journal.pone.0196032)
Supplement: S4 Fig — Box-whisker plots reflect medians, quartiles and outliers by lake/stream. (PDF) [file pone.0196032.s004.pdf]

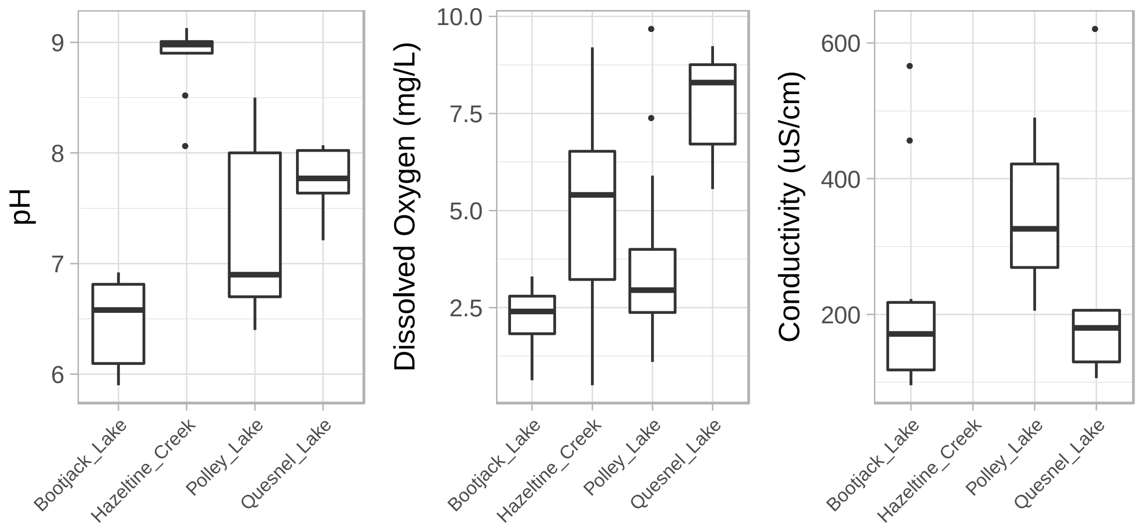

S4 Figure. Pore water pH, dissolved oxygen (mg/L) and conductivity (µS/cm). Box-whisker plots reflect medians, quartiles and outliers by lake/stream.
