# Supplementary material for: Short-term microbial effects of a large-scale mine-tailing storage facility collapse on the local natural environment
Source: PLoS One. 2018 Apr 25;13(4):e0196032. doi: 10.1371/journal.pone.0196032 (PMC5918821; doi:10.1371/journal.pone.0196032)
Supplement: S5 Fig — Point size reflects relative species richness (estimated as number of distinct OTUs). (PDF) [file pone.0196032.s005.pdf]

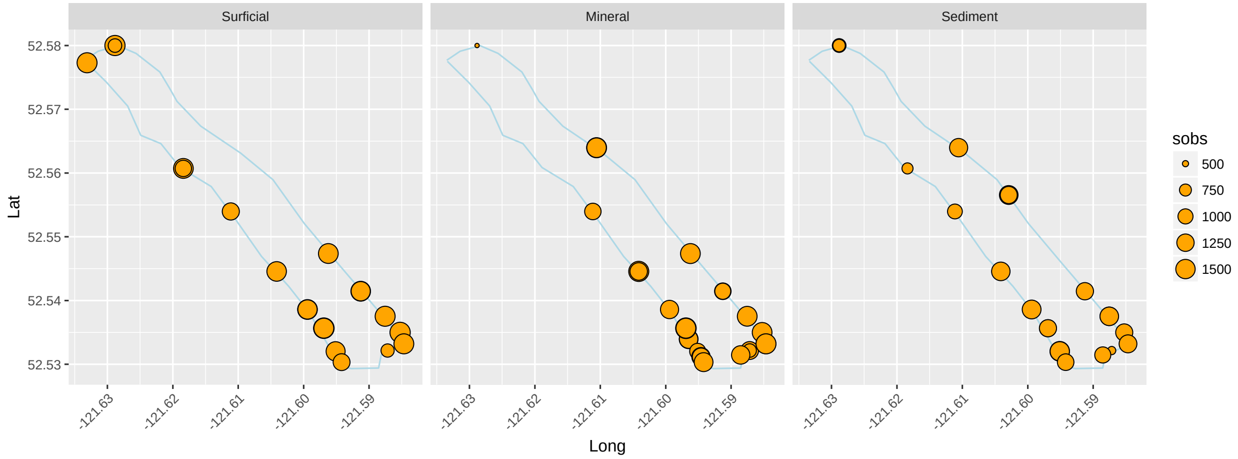

S5 Figure. Species richness (sobs) in Polley Lake microbiomes. Point size reflects relative species richness (estimated as number of distinct OTUs).
