# Supplementary material for: Short-term microbial effects of a large-scale mine-tailing storage facility collapse on the local natural environment
Source: PLoS One. 2018 Apr 25;13(4):e0196032. doi: 10.1371/journal.pone.0196032 (PMC5918821; doi:10.1371/journal.pone.0196032)
Supplement: S6 Fig — Relative abundance (% of total bacterial read counts) is expressed for the three sample types. (PDF) [file pone.0196032.s006.pdf]

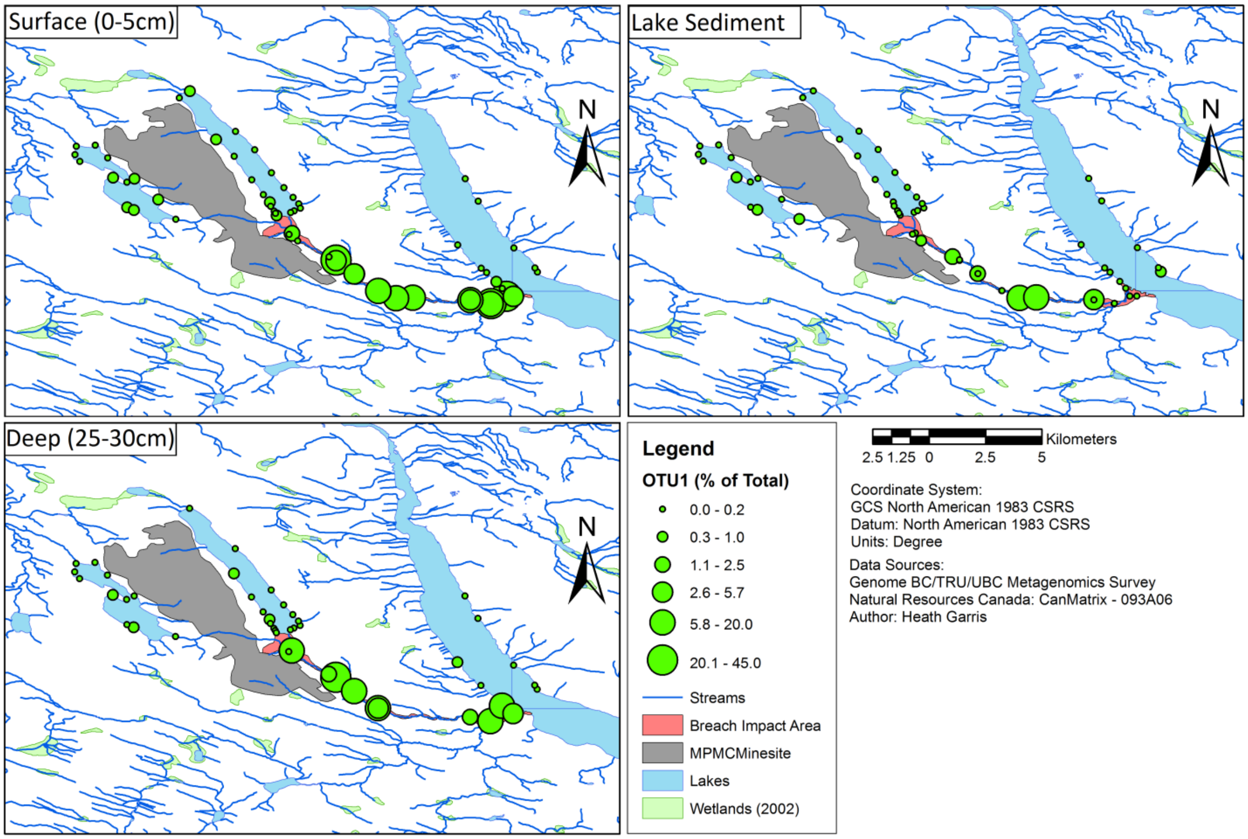

S6 Figure. Landscape-scale distribution of OTU1. Relative abundance (% of total bacterial read counts) is expressed for the three sample types.
