# Supplementary material for: Short-term microbial effects of a large-scale mine-tailing storage facility collapse on the local natural environment
Source: PLoS One. 2018 Apr 25;13(4):e0196032. doi: 10.1371/journal.pone.0196032 (PMC5918821; doi:10.1371/journal.pone.0196032)
Supplement: S1 Table — (PDF) [file pone.0196032.s007.pdf]

S1 Table. **Transect origination coordinates** are provided below for the 60 sites (in decimal degree format) with date, local (Pacific Standard) time of day (TOD) and site ID codes for the biomonitoring network.

DATE	TOD	LAT/LONG (°)	ID	DATE	TOD	LAT/LONG (°)	ID
6-Oct	11:45	N52.57999, W121.62884	1A	8-Oct	15:34	N52.54543, W121.65953	31A
6-Oct	12:41	N52.56072, W121.61839	2A	8-Oct	16:05	N52.54359, W121.65402	32A
6-Oct	13:44	N52.55397, W121.61114	3A	8-Oct	16:28	N52.54499, W121.65102	33A
6-Oct	14:20	N52.54457, W121.60412	4A	21-Oct	9:34	N52.51374, W121.57317	34A
6-Oct	14:56	N52.53860, W121.59941	5A	21-Oct	10:29	N52.51228, W121.57035	35A
6-Oct	15:28	N52.53566, W121.59691	6A	21-Oct	10:55	N52.51247, W121.57034	36A
6-Oct	15:55	N52.53753, W121.58754	7A	21-Oct	12:30	N52.49656, W121.51662	37A
6-Oct	16:24	N52.53500, W121.58525	8A	21-Oct	13:54	N52.49495, W121.50851	38A
6-Oct	16:45	N52.53321, W121.58467	9A	21-Oct	12:59	N52.49648, W121.51669	39A
6-Oct	17:10	N52.53216, W121.58716	10A	21-Oct	14:15	N52.49505, W121.50841	40A
6-Oct	17:30	N52.53147, W121.58853	11A	21-Oct	15:32	N52.50016, W121.55354	41A
7-Oct	11:00	N52.50383, W121.50623	12A	21-Oct	15:54	N52.50021, W121.55341	42A
7-Oct	10:16	N52.49801, W121.49944	13A	22-Oct	9:19	N52.50115, W121.50394	43A
7-Oct	11:34	N52.50768, W121.48987	14A	22-Oct	10:02	N52.49797, W121.50229	44A
7-Oct	12:46	N52.50932, W121.49087	15A	22-Oct	11:30	N52.49749, W121.53968	45A
7-Oct	13:15	N52.50753, W121.51014	16A	22-Oct	12:04	N52.49722, W121.54636	46A
7-Oct	13:43	N52.50926, W121.51233	17A	22-Oct	13:15	N52.50695, W121.56316	47A
7-Oct	14:28	N52.51730, W121.49928	18A	22-Oct	13:36	N52.50697, W121.56307	48A
7-Oct	14:56	N52.51855, W121.52177	19A	22-Oct	14:55	N52.52316, W121.58804	49A
7-Oct	15:28	N52.53609, W121.51356	20A	22-Oct	15:08	N52.52278, W121.58916	50A
7-Oct	16:02	N52.54501, W121.51871	21A	22-Oct	15:59	N52.52017, W121.58575	51A
8-Oct	10:56	N52.55796, W121.67427	22A	23-Oct	11:48	N52.57729, W121.63311	52A
8-Oct	11:20	N52.55846, W121.66669	23A	23-Oct	12:21	N52.56397, W121.61057	53A
8-Oct	11:48	N52.55463, W121.67474	24A	23-Oct	12:53	N52.55655, W121.60291	54A
8-Oct	12:20	N52.55198, W121.67287	25A	23-Oct	13:35	N52.54737, W121.59622	55A
8-Oct	13:02	N52.55328, W121.66171	26A	23-Oct	14:00	N52.54146, W121.59127	56A
8-Oct	13:40	N52.53669, W121.64145	27A	23-Oct	14:34	N52.53395, W121.59649	57A
8-Oct	14:20	N52.52885, W121.63452	28A	23-Oct	14:54	N52.53203, W121.59511	58A
8-Oct	14:30	N52.53242, W121.65123	29A	23-Oct	15:20	N52.53119, W121.59460	59A
8-Oct	15:03	N52.53355, W121.65391	30A	23-Oct	15:45	N52.53034, W121.59420	60A
