# Supplementary material for: Short-term microbial effects of a large-scale mine-tailing storage facility collapse on the local natural environment
Source: PLoS One. 2018 Apr 25;13(4):e0196032. doi: 10.1371/journal.pone.0196032 (PMC5918821; doi:10.1371/journal.pone.0196032)
Supplement: S2 Table — Fields in grey marked with (—) yielded insufficient data for reliable comparisons (<4 observations). (PDF) [file pone.0196032.s008.pdf]

S2 Table. **Water Quality Metric Means ± Standard Error**. Fields in grey marked with (--) yielded insufficient data for reliable comparisons (<4 observations).

	Bootjack Lake		Polley Lake		Hazeltine Creek		Quesnel Lake	
**Parameter**	**Surface**	**Porewater**	**Surface**	**Porewater**	**Surface**	**Porewater**	**Surface**	**Porewater**
Temperature (°C)	12.6±0.2	11.9±0.4	11.3±0.4	11.1±0.5	8.6±0.4	16.7±7.3	13.5±0.1	14.4±0.5
Conductivity (µS/cm)	75.3±0.6	211.9±42.9	186.5±2.8	343.2±31.5	--	--	106.3±0.1	236±79
Total Dissolved Solids (g/L)	--	--	0.2±0	0.2±0.1	8.2±8.8	0.2±0.1	--	--
Salinity (SAL)	0.1±0	0.1±0	0.1±0	0.2±0	0.2±0	0.2±0.1	--	--
DO (%)	90.2±1	21±2.3	82.1±5.6	33±5	96±5.5	43.8±6.7	--	--
DO (mg/L)	9.6±0.1	4.7±2.6	9.1±0.5	3.5±0.5	11.1±0.7	5±0.8	9.8±0	7.7±0.6
pH (pH)	7.4±0.1	6.5±0.1	8.6±0.1	7.3±0.2	9±0.1	8.9±0.1	8.3±0.2	7.8±0.1
pHmV (pHmV)	-44.2±4.6	0.7±14.4	-105.8±3.4	4±4.5	--	--	--	--
ORP (mV)	--	--	14.5±27.4	-52±19.8	116±12.2	111.3±16.5	--	--
Pressure (mmHg)	679.3±0.8	678.1±0.1	683.6±0	683.6±0	--	674±0.8	--	--
Turbidity (NTU)	18.4±13.3	1238±145	8±2.8	1889±369	--	--	--	--
Nitrate*	--	0.33±0.05	0.22±0.06	0.25±0.1	0.45±0.12	2.34±1.97	0.23±0.08	1.35±0.73
Phosphorus*	--	0.03±0.01	0.06±0.01	0.04±0.01	0.06±0.01	0.03±0.02	0.03±0.01	0.03±0.02
Ammonium*	--	0.34±0.13	0.34±0.15	1±0.38	0.15±0.04	0.03±0.03	0.01±0.01	0.1±0.08
Sulfate*	--	--	95.09±10.54	124.78±21.13	120.53±24.59	250.57±91.1	--	--
Sulfide*†	--	--	0±0	0±0	0±0	0±0	--	--
