# Supplementary material for: Short-term microbial effects of a large-scale mine-tailing storage facility collapse on the local natural environment
Source: PLoS One. 2018 Apr 25;13(4):e0196032. doi: 10.1371/journal.pone.0196032 (PMC5918821; doi:10.1371/journal.pone.0196032)
Supplement: S3 Table — Major water bodies are labelled as follows: BJL = Bootjack Lake, HAZ = Hazeltine Creek, POL = Polley Lake, QUE = Quesnel Lake. (PDF) [file pone.0196032.s009.pdf]

**S3 Table. Chemistry of the sediment layer pore water**. Major water bodies are labelled as follows : BJL = Bootjack Lake, HAZ = Hazeltine Creek, POL = Polley Lake, QUE = Quesnel Lake.

| Lake | SiteID | $SO_4^{2-}$ | $NH_4^+$ | $PO_4^{3-}$ | $NO_3^-$ |
|---|---|---|---|---|---|
| BJL | 22A | na | 0.1 | 0 | 0.22 |
| BJL | 23A | na | 0.2 | 0 | 0.1 |
| BJL | 24A | na | 0 | 0 | 0.3 |
| BJL | 25A | na | 0.4 | 0 | 0.43 |
| BJL | 26A | na | 0.1 | 0.06 | 0 |
| BJL | 27A | na | 0.1 | 0 | 0.42 |
| BJL | 28A | na | 0.6 | 0 | 0.24 |
| BJL | 29A | na | na | na | na |
| BJL | 30A | na | 0.2 | 0.04 | 0.44 |
| BJL | 31A | na | 0.3 | 0.05 | 0.53 |
| BJL | 32A | na | 1.5 | 0.07 | 0.46 |
| BJL | 33A | na | 0.2 | 0.09 | 0.46 |
| HAZ | 36A | 940 | 0 | 0.05 | 8.24 |
| HAZ | 37A | 85 | 0.1 | 0 | 0.1 |
| HAZ | 41A | 154 | na | na | na |
| HAZ | 42A | 73 | 0 | 0 | 0.27 |
| HAZ | 43A | 291 | na | na | na |
| HAZ | 45A | 177 | na | na | na |
| HAZ | 46A | 140 | na | na | na |
| HAZ | 50A | 317 | na | na | na |
| HAZ | 51A | 79 | 0 | 0.06 | 0.75 |
| POL | 1A | na | 1.5 | 0.08 | 0.35 |
| POL | 2A | na | 0.4 | 0.07 | 0 |
| POL | 3A | na | 0.5 | 0.08 | 0 |
| POL | 4A | na | 0.5 | 0.08 | 0.3 |
| POL | 5A | na | 0.3 | 0 | 0.21 |
| POL | 6A | na | 0.7 | 0.04 | 0.27 |
| POL | 7A | na | 1 | 0 | 0.21 |
| POL | 8A | na | 0.7 | 0.05 | 0.26 |
| POL | 9A | na | 0.8 | 0.06 | 0.21 |
| POL | 10A | na | 0.3 | 0 | 0.23 |
| POL | 11A | na | 1 | 0.08 | 0.27 |
| POL | 52A | 74 | 0 | 0.04 | 0 |
| POL | 53A | 172 | 0.2 | 0.07 | 2.03 |
| POL | 54A | 88 | 0.1 | 0 | 0 |
| POL | 55A | 95 | 0.1 | 0 | 0.35 |
| POL | 56A | na | 0.3 | 0.04 | 0 |
| POL | 57A | 85 | 2 | 0.07 | 0 |
| POL | 58A | 206 | 0.6 | 0 | 0 |
| POL | 59A | 71 | 1 | 0.06 | 0.34 |
| POL | 60A | 207 | 8 | 0.06 | 0 |
| QUE | 12A | na | 0 | 0 | 4.11 |
| QUE | 16A | na | 0 | 0.11 | 1.28 |
| QUE | 18A | na | 0.1 | 0 | 1.15 |
| QUE | 19A | na | 0 | 0.06 | 0 |
| QUE | 21A | na | 0.4 | 0 | 0.23 |

| Lake | Metric | $SO_4^{2-}$ | $NH_4^+$ | $PO_4^{3-}$ | $NO_3^-$ |
|---|---|---|---|---|---|
| BJL | Ave | | 0.3 | 0.03 | 0.33 |
| | Std-dev | | 0.4 | 0.03 | 0.16 |
| HAZ | Ave | 251 | 0.03 | 0.03 | 2.34 |
| | Std-dev | 258 | 0.04 | 0.03 | 3.41 |
| POL | Ave | 125 | 1 | 0.04 | 0.25 |
| | Std-dev | 56 | 1.7 | 0.03 | 0.43 |
| QUE | Ave | | 0.1 | 0.03 | 1.35 |
| | Std-dev | | 0.2 | 0.05 | 1.47 |
